# Invaders taking over—Mollusc faunal change in volcanic barrier lakes of the Albertine Rift biodiversity hotspot

**DOI:** 10.1371/journal.pone.0352648

**Published:** 2026-06-30

**Authors:** Francis Ssenkuba, Marcellin Rwibutso, Marie Claire Dusabe, Julius Tumusiime, Christian Albrecht

**Affiliations:** 1 Department of Biology, Mbarara University of Science and Technology, Mbarara, Uganda; 2 Department of Animal Ecology and Systematics, Justus Liebig University Giessen, Germany, Giessen, Germany; 3 Center for International Development and Environmental Research (ZEU), Justus Liebig University Giessen, Giessen, Germany; Charles University: Univerzita Karlova, CZECHIA

## Abstract

East African freshwater are renowned biodiversity hotspots, particularly within the Rift Valley lakes, which exhibit exceptional species richness and endemism. However, research in this region has predominantly focused on large Rift lakes, with smaller volcanic barrier lakes remaining underexplored. This study integrated morphological, DNA barcoding, and ecological analyses to assess mollusc diversity, biogeographical affinities, environmental correlates of distribution, and community differences in lakes Bunyonyi, Mutanda, and Mulehe in Uganda, and Lakes Ruhondo and Burera in Rwanda. Results revealed a relatively high malacofaunal diversity in these lakes compared to some Great Lakes in the region, although these comparisons should be interpreted cautiously because sampling scope differs among studies. Assemblages largely exhibited Nilotic affinities and were dominated by widespread mollusc species. Our findings suggest ongoing homogenisation of mollusc communities, as indicated by reduced dispersion among invaded lakes. While this pattern is consistent with potential influences of anthropogenic activities and invasive species such as the North American crayfish (*Procambarus clarkii*), *Physella acuta*, and the Asian lineage of *Melanoides tuberculata*, our analyses do not allow us to disentangle invasion effects from other confounding factors, including lake-specific characteristics and environmental gradients*.* Therefore, invasion-driven homogenisation should be interpreted with caution. Nevertheless, this trend, coupled with habitat degradation from agriculture, pollution, and infrastructure development, may pose significant threats to the mollusc diversity in these lakes. This research underscores the importance of prioritising these overlooked, fragile ecosystems in the regional biodiversity conservation strategies to safeguard habitats for molluscs and other faunal elements. A comprehensive regional freshwater survey, including metabarcoding, is needed to document molluscan diversity patterns under ongoing environmental change and increasing anthropogenic pressure.

## Introduction

The freshwaters of Eastern Africa are globally recognised for their high levels of species richness and endemism, especially within the Rift Valley lakes [1]. In the East African Rift Valley, research on aquatic biodiversity assessment and conservation has focused on the large rift lakes, whereas small volcanic barrier lakes have received considerably less attention. Similarly, limnology research efforts have disproportionately focused on the large Rift lakes [2].

Lakes Mutanda, Bunyonyi, Mulehe, Ruhondo, and Burera are volcanic barrier lakes in the western arm of the East African Rift Valley [3]. Lakes Mutanda, Bunyonyi, and Mulehe, in Southwestern Uganda, together with lakes Ruhondo and Burera in Northern Rwanda, were formed during volcanic events in the western arm of the Rift Valley in the late Pleistocene period ~ 18,000 B.P [3,4]. This geological event drastically altered the region’s hydrology, creating new waterbodies [3,5]. Previously, the Lake Edward Basin’s southern drainage had relied on watersheds near present-day Bukavu, Democratic Republic of Congo, and the western slopes of the Rwanda and Kigezi Highlands. The Virunga volcanoes erupted, released lava and ash that blocked streams from these highlands about 100 km south of Lake Edward [3]. This led to flooding that formed the present-day lakes Ruhondo and Burera, which overflow southward and drain into Lake Victoria through the Nyabarango and Kagera Rivers, thereby joining the Nile Basin [3]. Simultaneously, the uppermost valleys of the eastern branch of the Rutshuru River were partly blocked by the stream of lava from a small isolated cluster of volcanoes at Muko, which led to the formation of Lake Bunyonyi [3]. Furthermore, this lava stream was obstructed at its middle course by the northern edge of the main lava field from the Virunga volcanoes behind which a valley was flooded, leading to the formation of Lake Mutanda and subsequently Lake Mulehe [3].

**Fig 1:**
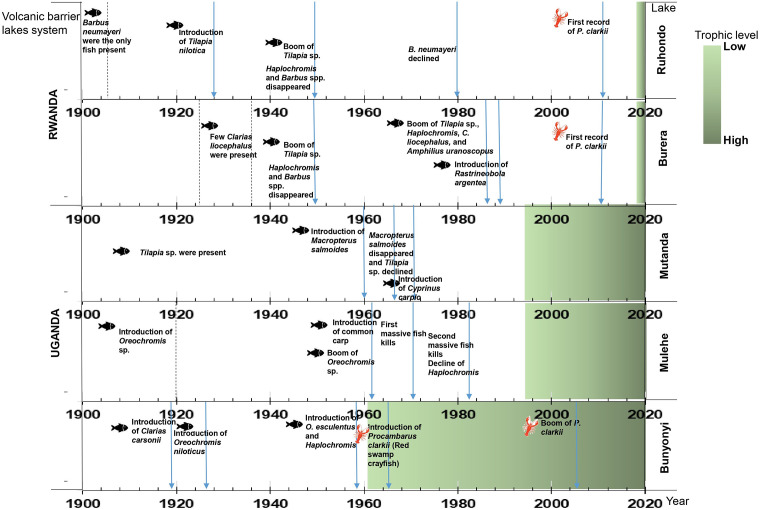
Summary of the major historical ecological events possibly causing significant ecosystem changes in the volcanic-barrier lakes (data from a review in S1 Table).

These lakes have for a long time experienced dramatic limnology and ecosystem shifts driven by natural and anthropogenic activities, especially the intentional introduction of alien species, which have caused detrimental effects on organismal communities, some of which are not fully understood (Fig 1; S1 Table).

The introduction of invasive species in an aquatic ecosystem can have disastrous effects on native aquatic biota. A notable example is the intentional introduction of the Nile Perch, *Lates niloticus* (Linnaeus, 1758), in Lake Victoria to boost commercial fisheries, which, combined with other invaders, e.g., water hyacinth, caused a massive ecosystem change, consequently resulting in massive extinction of endemic cichlid fishes [5,6]. However, invasive mollusc species are not known to pose a threat to Africa’s native molluscan fauna, with only localised impacts reported [7,8]. In contrast, their effects have been more pronounced in freshwater ecosystems across Eurasia, Europe, and the Americas [9]. However, an agonistic interaction such as inter- and intra-specific competition is a potential threat that has been observed among the native and alien invading molluscs of the Western Rift African Great Lakes [10]. In 1980, an Asian lineage *Melanoides tuberculata* (O. F. Müller, 1774) invaded Lake Malawi and later dispersed and invaded the ancient Lake Tanganyika, the Congo River, and continuously ascended into the Nile basin. A recently observed negative spatial correlation between the invasive Asian and native *M. tuberculata* morphs in Lake Malawi suggests an agonistic competitive interaction between the two lineages [6,10]. This has led to the displacement of the native morphs by the Asian morph in the southern part of the lake [11], potentially leading to genetic homogenisation, posing a threat to the conservation of the endemic gastropod diversity. This devastating threat is consistent with anthropogenic stressors that facilitate the invaders’ colonisation [7,12].

Similarly, two other invasive gastropods, pulmonates, *Pseudosuccinea columella* (Say, 1817) and *Physella acuta* Draparnaud, 1805, were introduced around 1940–1950 in the Republic of South Africa via the aquarium trade [13], and subsequently into the Albertine rift. *Physella acuta* has recently been encountered only in tributaries of an Albertine volcanic Lake Kivu by Dusabe et al. [14] but never in the lake proper. Nonetheless, *P. acuta* is predicted to continue its global invasion, including in Africa [15].

One notable introduction of an invasive species in the volcanic barrier lakes is that of the North American crayfish (*Procambarus clarkii* (Girard, 1852)) [16]. The species is reported to impact the native freshwater biodiversity negatively, especially the macroinvertebrates, due to its intensive predatory behaviour and high fecundity [17]. Furthermore, its tolerance to a wide range of environmental conditions, large size, and omnivorous feeding behaviour have facilitated its rapid establishment [17,18], potentially increasing competitive and predatory pressure on native fauna. The snail-feeding preference of *P. clarkii* has been evidenced in Kenya and Egypt based on laboratory experiments and field observations [19–21]. This non-native invasive crayfish was introduced in Lake Bunyonyi and was reported to be well-established and abundant as early as 2007 [22]. The widespread occurrence of the species within the Bunyonyi system indicates potential colonisation of lakes Mutanda and Mulehe, adjacent to Lake Bunyonyi. In Rwanda, little has been reported about the spread and establishment of *P. clarkii* except for its first introduction in Mukungwa Valley as a biological control agent of water hyacinth [18]. Exploring the threat this species poses could provide substantial insights into combating species loss, potentially mitigating expected mollusc diversity loss by guiding control efforts.

The establishment of *P. clarkii* in Lake Bunyonyi and its potential spread to adjacent lakes represents a concern for native mollusc populations, including endemic and conservation-priority species such as *Bulinus mutandaensis* (Preston, 1913) and *Gyraulus costulatus exilis*. These conservation-priority species are thought to be endemic to the lakes Mutanda and Bunyonyi system. *B. mutandaensis* has been treated as a distinct species by Mandahl-Barth [23], though considered to be a lacustrine form of *Bulinus truncatus* (Audouin, 1827) [24,25]. Furthermore, *G. costulatus exilis* has been considered a subspecies of *Gyraulus costulatus* (Krauss, 1848) by Mandahl-Barth [23], while considered morphologically indistinguishable from *G. costulatus* by Brown and Wright [24]. The taxonomic uncertainty about these species has remained unresolved for a long time based on morphological, anatomical, conchological, and ploidy level (*B. mutandaensis*) characteristics. It is possible that the currently observed shell variations in *B. mutandaensis* and *G. costulatus* might represent ecophenotypic plasticity. However, DNA barcoding may help to clarify this long-standing taxonomic uncertainty.

Currently, there have been very few surveys of macrobenthos, including molluscs, in these satellite volcanic barrier lakes. Most of what we know about the malacofauna in these lakes comes from the early expeditions published in [26] and [27], conducted in 1907 and 1931, respectively. Given this knowledge vacuum for almost 100 years, this study seeks to update our knowledge of the malacofaunal assemblage of these lakes.

Based on an integrated approach using morphological, DNA barcoding, and ecological analyses of mollusc communities, this study 1) determines the diversity and distribution of freshwater molluscs in lakes Bunyonyi, Mutanda, Mulehe, Ruhondo and Burera in the Western Rift valley region, 2) quantifies ecological factors driving molluscan distribution, 3) tests faunal change, and 4) discusses the biogeographical affinity of molluscs in the lakes.

We also discuss potential threats to the mollusc diversity and suggest conservation and management interventions.

## Materials and methods

### Study area

**Fig 2:**
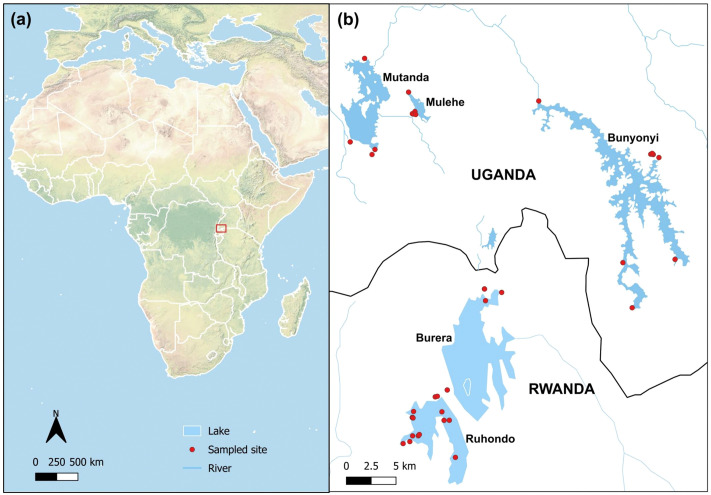
Map of the study area highlighting the sampled sites. (**a**) Map of Africa highlighting the region of the sampled volcanic barrier lakes (in a red frame) (**b**) Map of the sampled volcanic barrier lakes showing the location of the sampled sites (red points).

The sampled volcanic barrier lakes are located in southwestern Uganda and northwestern Rwanda (Fig 2). The Ugandan lakes are Lake Mutanda (1.179° S, 29.698° E, 1,792 m) with a total surface area of 29 km^2^ and a maximum depth of 56 m. Lake Bunyonyi (1.283° S, 29.927° E, 1,973 m) covers 56 km^2^ and has a maximum depth of 40 m. Lake Mulehe (1°13′5“ S, 29°43′17” E, 1,806m) has a surface area of 5km^2^ and a maximum depth of 6 m [4]. In northwestern Rwanda, the lakes are Lake Ruhondo (1.534° S, 29.711° E, 1,765 m) with a surface area of 26.6 km^2^ and a maximum depth of 68 m. Lake Burera (1.45° S, 29.78° E, 1,860 m) spans 51.8 km^2^ and reaches depth of 179 m [28]. They are all located near the foot of the Virunga mountain range [4].

The Ugandan volcanic barrier lakes are connected by several adjoining streams and rivers for examples, lakes Mutanda and Mulehe are linked by River Mucha, which is approximately 2 km long. Lake Bunyonyi is connected to Lake Mutanda by the River Ruhezamyenda, and Lake Mutanda drains into River Mkarara, which drains into Lake Edward [16,29]. The lakes’ catchments are dominated by cultivated hills, with the lakes’ riparian zones increasingly encroached on by agricultural activities and hotel development [29]. Lakes Ruhondo and Burera are hydrologically interconnected through short streams and underground watercourses due to their proximity and difference in elevation. Both lakes drain into the Nile Basin, with their outflow directed through the Mukungwa River, which flows southward via Lake Ruhondo. The Mukungwa River subsequently joins the Nyabarongo River, which, after converging with the Ruvubu River from Burundi, forms the Kagera River, ultimately discharging into Lake Victoria [28,30].

The study area is situated in a predominantly agricultural region, characterised by intensive farming practices [31]. The surrounding landscape features extensive human settlement, infrastructure development, and hydropower activities, including the Ntaruka Power Station [28]. These anthropogenic activities pose significant threats to the ecological integrity of lakes Ruhondo, Burera, Mutanda, Mulehe, and Bunyonyi, including sedimentation, agrochemical runoff, and sewage pollution.

### Sampling

Extensive malacological surveys were conducted in 2023 and 2024 in lakes Bunyonyi, Mutanda, Mulehe, Ruhondo, and Burera. A total of 44 sites were comprehensively and purposively sampled (Fig 1), taking into account the spatial distribution of the lake sites and the diversity of available microhabitats. Snails were collected using a scoop net (20 cm diameter, 1 mm mesh size) and a Surber net (250 µm mesh size) with a standardised sampling effort of 30 minutes per site. Samples from stones and rocky substrates were collected by direct picking using feather-light forceps. An Ekman grab was used to collect samples from deeper littoral sites up to 20 m depth. Crayfish traps were set with bait (cat food and bacon) to catch *P. clarkii* in the littoral zone. Rarefaction curves were generated to assess sampling completeness and to standardise species richness across samples. Individual-based rarefaction and extrapolation were performed using the iNEXT package in R. Curves were constructed from species abundance data, with 95% confidence intervals estimated via a 50-times bootstrap resampling, though sites with very low counts were eliminated (see S7 Fig). At each site, selected physicochemical parameters, including dissolved oxygen (DO), water temperature, total dissolved solids (TDS), electrical conductivity (EC), and pH, were measured *in situ* using a calibrated handheld multiparameter probe meter (HI 9829, HANNA, Romania). GPS coordinates and altitude were recorded using a portable GPS (Garmin GPSMAP 64), following the manufacturer’s guidelines (S3 Table). Furthermore, we documented site use and substrate types (vegetation, rock, sand, sapropel, detritus). All collected samples were preserved in 80% ethanol and appropriately labelled. Specimen vouchers are stored in the Systematics and Biodiversity collection of the Justus Liebig University, Giessen (UGSB). Specimens were identified to genus and species level based on shell morphology [25,32–34]. Two specimens per species and locality were photographed with a Keyence digital microscope (KEYENCE VHX-2000, Keyence Deutschland GmbH, Neu-Isenburg, Germany) before processing in the molecular laboratory.

### DNA extraction, amplification and sequence analysis

A total of 80 specimens representing the genera: *Biomphalaria*, *Bulinus*, *Afrogyrorbis*, *Sphaerium*, *Radix*, *Pisidium*, *Physella*, *Melanoides*, *Bellamya*, *Segmentorbis*, *Gyraulus*, and *Procambarus* were selected for DNA sequencing to resolve taxonomic uncertainties. Genomic DNA was extracted from a small piece of foot muscle for molluscs and from the abdomen for *P. clarkii* using the CTAB protocol [35]. The mitochondrial cytochrome *c* oxidase subunit I (COI) and large subunit ribosomal RNA (16S rRNA) gene fragments were amplified using primers COF14 and COR722b [36] for COI and 16Sar and 16Sbr for 16S [37]. Polymerase chain reaction conditions were applied as described by Dusabe et al. [14]. Sequencing was performed using the BigDye Terminator Kit on an ABI 3730xl DNA analyser (Life Technologies, LGC Genomics GmbH). The new sequences are deposited in NCBI GenBank (accession numbers are provided in S2 Table). Sequence editing was performed manually and aligned using BioEdit v7.2 [38].

Edited sequences were analysed using BLASTn (megablast) against the NCBI GenBank nucleotide database. BLAST results were ranked by maximum score, and the top two hits for each specimen were selected for further comparison. The best match among these hits was identified based on the lowest E-value. For Sphaeriidae*,* local BLAST searches were additionally conducted against an internal sequence database (unpublished data), as closely related sequences were not available in NCBI GenBank. These assignments should therefore be regarded as provisional pending broader taxonomic revision.

### Phylogenetic and phylogeographical analyses

A phylogenetic analysis was performed only for *Bulinus*, *Biomphalaria*, and *Melanoides* species using the COI marker to study the taxonomic status of *Bulinus mutandaensis*, to address the *Biomphalaria pfeifferi* (Krauss, 1848) and *B*. *choanomphala* species delimitation problem, and to ascertain whether the recorded *M. tuberculata* is of the invasive Asian or the native lineage (S2 Fig). For *Bulinus* sp., a total of 47 in-group specimens were included, and *B. pfeifferi* was used as the outgroup. For *Biomphalaria* sp., a total of 19 in-group specimens were included, and *Biomphalaria glabrata* was used as an outgroup (S3 Fig). For *Melanoides* sp., a total of 74 in-group specimens were included, and *Hydrobia glyca* and *Lavigeria grandis* were used as outgroups (S2 Fig). The *Melanoides* sp. dataset used was adopted from [10]. Sequence alignments were performed using ClustalW implemented in BioEdit. The final alignment lengths differed among taxa due to trimming of low-quality sequence ends and the retention of only overlapping regions across all sequences within each taxonomic group. The resulting alignment lengths were 600 bp for *Bulinus* spp., 588 bp for *Biomphalaria* spp., 580 bp for *Melanoides* spp. (COI), and 502 bp for bivalves (16S rRNA). The best-fit substitution model was selected in MEGA v11 based on the Akaike Information Criterion (AIC) and the Bayesian Information Criterion (BIC). Maximum-likelihood trees were then constructed using the Hasegawa-Kishino-Yano model with 1,000 bootstrap replicates.

Statistical parsimony haplotype networks were constructed in PopArt v1.7 [39] to evaluate haplotype diversity among *Bulinus* specimens from the study lakes and to potentially clarify the taxonomic status of *Bulinus mutandaensis* (S2 Fig).

### Mollusc diversity and ecological data analyses

Molluscan diversity was calculated using the Shannon-Wiener (H) index in PAST (Version 3.15) for each sampled site. The H-index values along with their coordinates were imported into QGIS software (Version 3.36.1) and thematically mapped using a graduated colour method to display the spatial distribution of mollusc species diversity in the studied lakes.

Non-metric Multidimensional Scaling (NMDS) was used to explore the variations in mollusc community composition across sampling sites among the sampled lakes. A Bray-Curtis dissimilarity matrix was calculated from Hellinger-transformed mollusc species abundance data. The NMDS ordination (k = 2 dimensions) was performed using the *metaMDS()* function in the vegan package of R Studio (version 4.3.3), resulting in a stress value of 0.058, indicating a reliable representation of the data.

Environmental variables, including altitude, physicochemical parameters (DO, pH, TDS, EC, and temperature), human activities (fishing, agriculture, boating, tourism, settlement, water sporting), substrate type (sand, sapropel, detritus, vegetation, and stones), and the system type (lake or stream) were considered in this study. Pairwise correlations among environmental variables revealed several highly correlated predictors (|r| > 0.7), including EC and TDS, substrate variables, and disturbance-related variables (S5 Fig). To reduce collinearity, TDS was excluded due to a very strong correlation with EC (r = 0.998), and only one variable from correlated groups (e.g., detritus vs. sand/vegetation; settlement vs. tourism, stream vs. lake) was retained for analysis. Altitude and EC were retained despite correlation, as they represent distinct ecological gradients, but were interpreted cautiously. Selected variables were fitted onto the ordination using the *envfit()* function. The categorical variables were analysed as presence/absence data (1/0). Vector significance was determined via permutation testing (999 permutations, p < 0.05). Significant group differences were confirmed using PERMANOVA in R Studio (version 4.3.3).

Beta-diversity among lakes was quantified using Bray-Curtis dissimilarity. Differences in community composition associated with invasion status and disturbance gradients were tested using PERMANOVA. Community homogenisation was evaluated using multivariate dispersion analysis (PERMDISP), which tests whether groups differ in their dispersion around centroids in multivariate space. All analyses were conducted in R using the *vegan* package with 999 permutations.

## Results

### Diversity and distribution of molluscs

**Fig 3:**
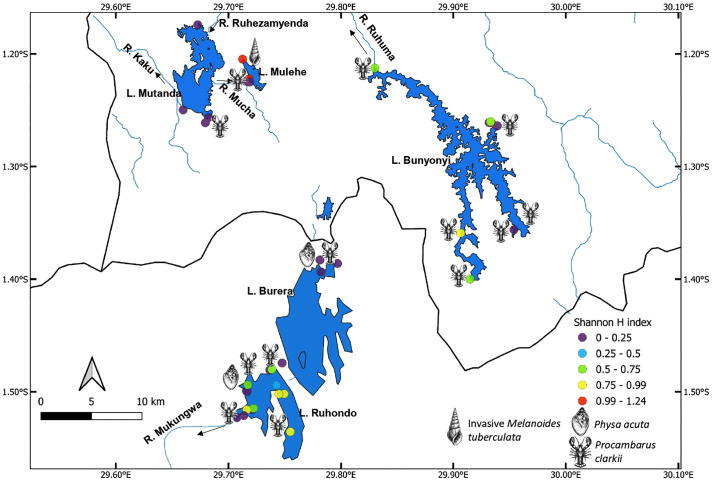
Mollusc diversity distribution (heatmap) and occurrence of the invasive Melanoides tuberculata lineage, Physella acuta, and Procambarus clarkii. Shannon H index represents mollusc diversity based on the Shannon-Wiener diversity index. Colour codes used for diversity groups: Purple – very low mollusc diversity site, Pale-blue – low mollusc diversity site, Pale-green – medium mollusc diversity site, Yellow – high mollusc diversity site, Red – very high mollusc diversity site. Illustrations of Physella acuta, Procambarus clarkii, and invasive Melanoides tuberculata were created by the authors for illustrative purposes and are not reproductions of previously published images.

We identified 18 mollusc species belonging to eight families, comprising 13 gastropod species and 5 bivalve species (Table 1). The highest mollusc diversities (H index = 0.99–1.24) were found in lakes Mulehe, Bunyonyi and Ruhondo (Fig 3). In contrast, lakes Mutanda and Burera exhibited the lowest mollusc diversity, ranging between 0 and 0.25 (Fig 3).

**Table 1:** Historical malacofauna versus species found in the current study. Current mollusc species found in lakes Bunyonyi (BN), Mutanda (MT), Mulehe (MH), Ruhondo (RH), and Burera (BR) are compared with the past reports by Worthington [27] in 1931, and Thiele [26] in 1907-1908, respectively. Synonyms adopted here refer to the names used in the original papers, i.e., [27] and [26]. Nomenclature follows MolluscaBase (https://www.molluscabase.org, 14^th^ January, 2024).

Species	Past (1907/1931)			Recent (2023/2024)				
	Synonym	MT-BN (1931) [27]	RH (1907) [26]	BN	MT	MH	RH	BR
**GASTROPODA**								
**Ampullariidae**								
*Pila ovata* (Olivier, 1804)						**X**		
**Bulinidae**								
Bulinus trigonus (E. Von Martens, 1892)	*Isidora strigosa*		**X**					
*Bulinus tropicus* (Krauss, 1848)		**X**					**X**	
*Bulinus truncatus* (Audouin, 1827)				**X**		**X**	**X**	
	*Bulinus tropicus mutandaensis*	**X**				**X**		
*Bulinus ugandae* Mandahl-Barth, 1954	*Bulinus globosus ugandae*	**X**						
*Bulinus zanzibaricus* (Clessin, 1886)*	*Isidora zanzibarica*		**X**					
**Lymnaeidae**								
*Radix natalensis* (Krauss, 1848)				**X**	**X**	**X**	**X**	**X**
**Physidae**								
*Physella acuta* Draparnaud, 1805							**X**	**X**
**Planorbidae**								
*Afrogyrorbis natalensis* (Krauss, 1848)	*Anisus natalensis*	**X**		**X**				
*Biomphalaria* cf. *choanomphala* (E. von Martens, 1879)				**X**	**X**	**X**		
*Biomphalaria pfeifferi* (Krauss, 1848)	*Biomphalaria ruppelli* *Planorbis nairobiensis*	**X**	**X**				**X**	
*Burnupia* sp.		**X**						
*Ferrissia* sp. Pettancylus ruandensis (Thiele, 1911)				**X**				
	*Ancylus ruandensis*		**X**					
*Gyraulus costulatus* (Krauss, 1848)	*Gyraulus costulatus exilis*	**X**		**X**				
*Segmentorbis* cf. *angustus* (Jickeli, 1874)	*Segmentorbis kempi*	**X**		**X**			**X**	
**Thiaridae**								
*Melanoides tuberculata* (O.F. Müller, 1774)				**X**		**X**	**X**	**X**
**Viviparidae**								
*Bellamya* cf. *unicolor* (Olivier, 1804)							**X**	
**BIVALVIA**								
**Sphaeriidae**								
*Euglesa* cf. *keniana* (Preston, 1911)				**X**				
*Euglesa* sp. I				**X**				
*Euglesa* sp. II				**X**				
*Euglesa* sp. III				**X**				
*Sphaerium* sp. (*Sphaerium nyanzae* E.A. Smith, 1892 for RH)		**X**	**X**	**X**			**X**	
**Total number**		**9**	**5**	**13**	**2**	**5**	**9**	**3**

*This species is only known from the original description and has not been considered in more recent taxonomic revisions of *Bulinus* spp.

*Pila ovata* (Olivier, 1804) was exclusively recorded from Lake Mulehe, where *Bulinus cf. mutandaensis* was also found. *Bulinus tropicus* (Krauss, 1848) was restricted to Lake Ruhondo. The globally invasive species *Physella acuta* was detected exclusively in lakes Burera and Ruhondo. *Afrogyrorbis natalensis* (Krauss, 1848) and *Segmentorbis* cf*. angustus* (Jickeli, 1874) were recorded solely from Lake Bunyonyi, whereas *Lentorbis* sp. was found only in Lake Ruhondo. *Bellamya* cf. *unicolor* (Olivier, 1804) was also confined to Lake Ruhondo. *Biomphalaria* cf. *choanomphala* (E. von Martens, 1879) was recorded in all three sampled lakes in Uganda, i.e., lakes Bunyonyi, Mulehe, and Mutanda. *Radix natalensis* (Krauss, 1848) and *M. tuberculata* were recorded in all five sampled lakes (Table 1). However, two distinct lineages of *M. tuberculata*, i.e., the indigenous and the invasive Asian morphs, were identified in the region (S2 Fig). The invasive Asian morph was detected exclusively in Lake Mulehe.

Sphaeriid bivalves were encountered exclusively in lakes Bunyonyi and Ruhondo (Table 1). The encountered species were assigned provisional working names (see S2 Table and S6 Fig for details). Notably, five out of the seven recorded sphaeriid species were found in Lake Bunyonyi. All *Euglesa* spp. were collected from tributaries supplying Lake Bunyonyi, whereas *Sphaerium* sp. was recorded from the lakes Bunyonyi and Ruhondo.

### Ecological factors driving the mollusc distribution

**Fig 4:**
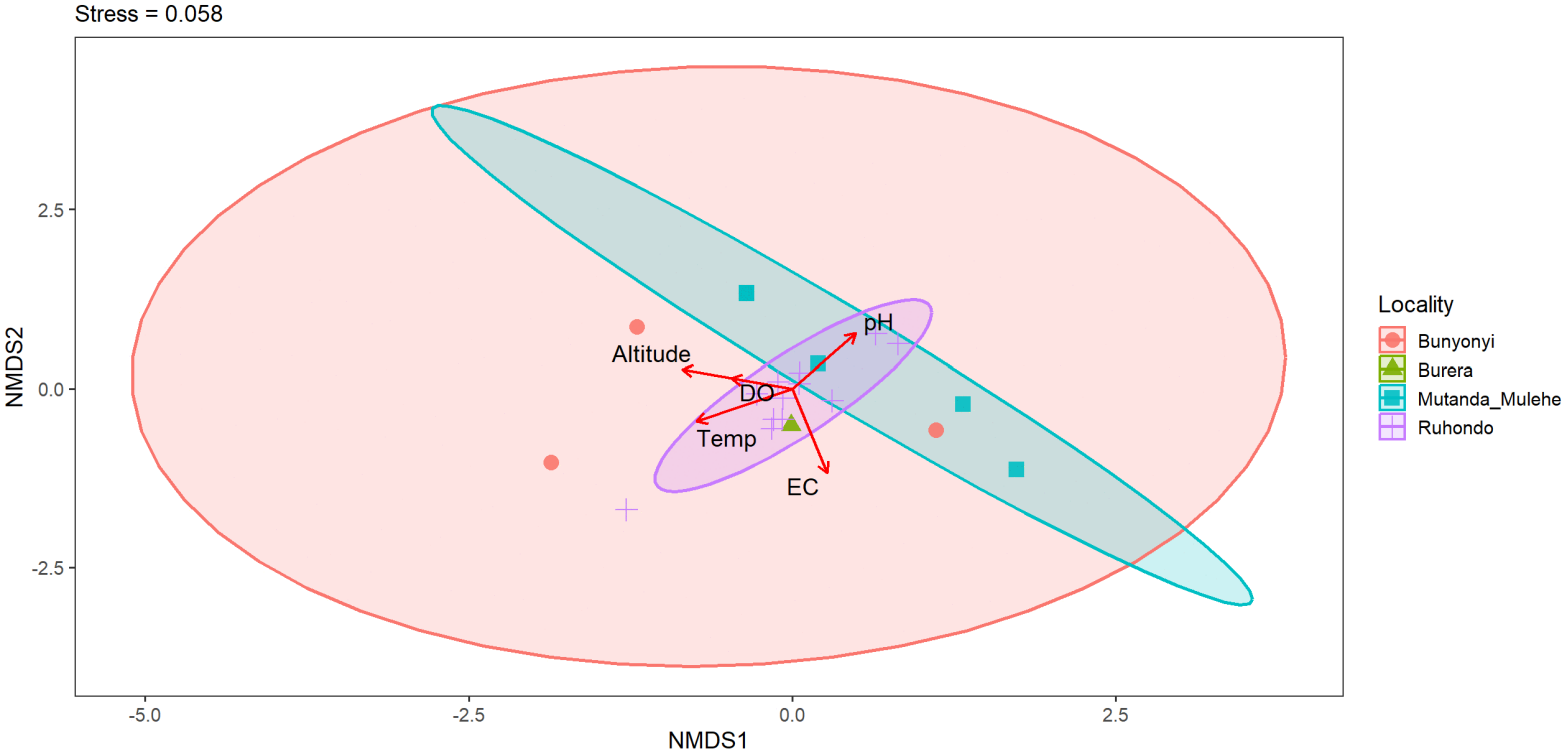
NMDS ordination of mollusc community composition explained by environmental parameters. Abbreviations: DO-Dissolved oxygen, EC-Electrical conductivity, Temp-Temperature.

The NMDS ordination biplot shows the variation in malacofaunal community composition among the sampling sites in the five lakes. The stress value of 0.058 suggests that the ordination adequately represents the data. PERMANOVA indicated that locality was significantly associated with community composition (pseudo-F = 1.971, R² = 0.25, p = 0.036). Samples from the Lake Mulehe-Mutanda system, and from lakes Ruhondo and Bunyonyi cluster together, indicating mollusc species composition similarity. Lake Bunyonyi and Lake Burera sites are widely separated with no overlap, indicating a significant dissimilarity among the mollusc communities of these lakes from the rest of the four lake systems. The environmental parameters, i.e., EC (p = 0.043), pH (p = 0.002), and fishing (p = 0.003), are significantly associated with mollusc community composition (Fig 4, S8 Fig).

The current study reports an extensive distribution of the invasive crayfish *P. clarkii* in lakes Bunyonyi, Ruhondo, and Burera (Fig 3). The invasion is ongoing, with early-stage establishment observed in lakes Mulehe and Mutanda. The invasive *M. tuberculata* was encountered from only one locality in Lake Mulehe (Fig 3). On the other hand, *P. acuta* was encountered in two lakes, i.e., Lakes Ruhondo and Burera (Fig 3).

Multivariate dispersion analysis based on Bray–Curtis dissimilarities revealed significant differences in community dispersion among invasion categories (PERMDISP permutation test: F = 9.03, p = 0.001). Lakes with invasive species showed lower mean distances to group centroids, indicating greater similarity in mollusc community composition among invaded systems, partially highlighting the ongoing mollusc community homogenisation.

In contrast, dispersion did not differ significantly among disturbance categories (PERMDISP permutation test: F = 2.37, p = 0.179), suggesting that anthropogenic disturbance alone did not lead to measurable homogenisation of communities.

### Changes in molluscan community composition in volcanic barrier lakes

A comparative analysis of historical records and our recent surveys suggests changes in the molluscan communities of the sampled volcanic barrier lakes over the past 90 years. Worthington [27] previously documented *B. tropicus*, *B. ugandae*, *B. pfeifferi*, and *Burnupia* sp. in lakes Bunyonyi, Mutanda, and Mulehe. However, our current survey found no evidence of these species in these lakes, suggesting population decline or potential local extirpation. Additionally, we document the first occurrences of *B. truncatus*, *M. tuberculata*, *E* cf. *keniana* (Preston, 1911), *P. ovata*, and *B.* cf*. choanomphala* in these lakes (Table 1), indicating a notable change in species composition.

On the other hand, Thiele [26] previously encountered B. trigonus, *B. zanzibaricus*, Pettancylus (*=*Ferrissia) ruandensis, and *S. nyanzae* in Lake Ruhondo and in the waterfalls between lakes Ruhondo and Burera (Table 1). It needs to be discussed whether these species conform to the *Bulinus*, *Ferrissia* and *Sphaerium* taxa encountered during our survey.

### Phylogenetic and phylogeographical analysis results

Our phylogenetic analysis revealed the presence of four well-supported *Bulinus* clades within the sampled volcanic barrier lakes, all clustering within the *B. truncatus/tropicus* complex (bootstrap support [BS] 0.81; S1 Fig (b)). Notably, *B* cf. *mutandaensis* formed a supported clade (BS 0.92) within this complex, clustering specifically with *B. truncatus* (S1 Fig (b)). Within our study area, we identified three haplotypes of *B. truncatus*, among which the *B.* cf. *mutandaensis* haplotype (UGSB 29948; PZ492956) was detected only in Lake Mulehe and its primary inflow, River Mucha (S1 Fig (a)). Thus, *B* cf. *mutandaensis* has been treated as *B. truncatus* in all ecological analyses.

Additionally, we identified two *M. tuberculata* lineages: an invasive Asian lineage in Lake Malawi (LMI) and a native African lineage (S2 Fig). The two *M. tuberculata* specimens from Lake Mulehe clustered within the Asian invasive lineage in Lake Malawi (LMI) and a Singapore lineage. In contrast, the specimens from Lake Bunyonyi grouped within the native *M. tuberculata* lineage (DR Congo, Lake Edward, CLE morph; S2 Fig).

### Biogeographical affinities

The molluscan fauna encountered in the volcanic barrier lakes exhibits a predominantly Nilotic affinity, dominated by species with broad Afrotropical distributions (Table 2).

**Table 2:** Biogeographical affinity of molluscs of the volcanic barrier lakes. The molluscs’ biogeographical affinities listed here are based on the phylogenetic analyses where the sequences are available and the respective standard literature.

Species		Phylogenetic relationship	Biogeographical affinity	Reference
**GASTROPODA**				
**Ampullariidae**				
*Pila ovata* (Olivier, 1804)		East Africa, Lake Victoria, Uganda lineage	Widespread throughout Africa	[25,40]
**Bulinidae**				
*Bulinus* cf. *mutandaensis* (Preston, 1913)		East Africa, Uganda lineage: in the group of *Bulinus truncatus*	Potentially endemic to Western Uganda crater lakes	[14,25,41]
*Bulinus tropicus* (Krauss, 1848)		East Africa, Uganda Crater lakes lineage	Widespread throughout Africa, but mainly in northern parts of the continent	[25,41]
*Bulinus truncatus* (Audouin, 1827)		East Africa, Rwanda lineage	Widespread throughout Africa, but mainly in northern parts of the continent	[14,25,41]
**Lymnaeidae**				
*Radix natalensis* (Krauss, 1848)		East Africa, Kenya and Rwanda lineage	Widespread in African freshwaters, potentially Asian affinities	[14,25,42]
**Physidae**				
*Physella acuta* Draparnaud, 1805		Invasive lineage	Widespread in Africa, Asia, North American affinities	[15,25]
**Planorbidae**				
*Afrogyrorbis natalensis* (Krauss, 1848)		n.a	East Africa, from Ethiopia to South Africa	[25]
*Biomphalaria* cf. *choanomphala* (E. von Martens, 1879)		East Africa, Rwanda lineage	Lake Kivu, lakes Victoria and Albert, Albert Nile	[14,25]
*Biomphalaria pfeifferi* (Krauss, 1848)		East Africa, Uganda, Kenya lineage	Tropical Africa	[14,25]
*Ferrissia* sp.		n.a	Genus widespread throughout Africa	[25]
*Lentorbis* sp.		n.a	Genus widespread in eastern and central Africa	[25,32]
*Segmentorbis* cf. *angustus* (Jickeli, 1874)		n.a	Tropical Africa	[25,32]
**Thiaridae**				
*Melanoides tuberculata* (O. F. Müller, 1774)		East Africa, Uganda, Rwanda, native lineage	Widespread in tropical Africa	[6,25]
*Melanoides tuberculata* (O. F. Müller, 1774)		Asian invasive lineage, China	Widespread in tropical Africa	[6]
**Viviparidae**				
*Bellamya* cf. *unicolor* (Olivier, 1804)		East Africa, Lake Victoria, Uganda lineage	Widespread throughout Africa	[25,43,44]
**BIVALVIA**				
**Sphaeriidae**				
*Euglesa* sp. I		n.a	n.a	
*Euglesa* sp. II		n.a	n.a	
*Euglesa* sp. III		n.a	n.a	
*Euglesa* cf. *keniana* (Preston, 1911)		n.a	Ethiopia to Zambia East African affinities	[34,45]
*Sphaerium* sp.		East Africa, Rwanda	Egypt, Ethiopia, to Northern Sudan, East Africa	[14,34]

## Discussion

### Diversity and distribution of molluscs

In general, the montane freshwater malacofauna of the East African Rift Valley system, including its crater and volcanic barrier lakes, has historically been considered species-poor [25,46]. From the Western Ugandan crater lakes, only eight gastropod species were reported, with *B. tropicus toroensis* and *Gabbiella kichwambae* considered endemic [25]. This low species richness has been attributed to unfavourable environmental conditions, including high salinity, low dissolved oxygen levels due to prolonged water residence time, and permanent stratification, all of which severely constrain mollusc survival [25].

Contrary to these earlier observations, the present study reveals a potentially comparatively richer malacofaunal assemblage within the volcanic barrier lakes cluster. Notably, Lakes Bunyonyi and Ruhondo exhibit higher molluscan species richness than some larger Western Rift Great Lakes. Species richness comparisons with other Western Rift lakes were based on published literature and therefore reflect differences in sampling scope and survey intensity among studies. These comparisons are intended to provide regional context rather than strictly standardised estimates of biodiversity. Lake Bunyonyi and its associated inflowing and outflowing tributaries yielded 13 mollusc species while Lake Ruhondo yielded nine molluscan species (Table 1), surpassing Lake Kivu, which contains about eight mollusc species according to recent assessments [14]. Lake Kivu also exhibits low molluscan diversity, with a Shannon diversity index ranging between 0 and 1.1. Similarly, Lakes George and Edward also have a lower recorded species richness of about 10 molluscs in the lake proper, and 15 mollusc species with the Kazinga channel species inclusive, respectively [25,32,47]. In contrast, some larger Western Rift lakes, such as Lake Albert, exhibit higher diversity, hosting at least 16 gastropod species, including eight endemics [25,32]. It is worth noting that no hydrobiid snails (*Gabbiella* spp.) were encountered in the sampled volcanic barrier lakes, which is consistent with earlier malacological surveys conducted in these systems. This observation is not unexpected, as hydrobiids are known to be highly sensitive to environmental disturbance and pollution [48]. In addition, many species within this family exhibit specific habitat requirements that may not be met in volcanic barrier lakes experiencing ongoing limnological changes [49].

The relatively high bivalve diversity disproportionate to the number of the sampled high-altitude volcanic barrier lakes in this study is not a surprise (see [45]). All *Euglesa* spp. (formerly under *Pisidium* spp.) were exclusively collected from tributaries supplying Lake Bunyonyi, whereas *Sphaerium* sp. was recorded in the lake proper of Lakes Bunyonyi and Ruhondo (Table 1). This distribution pattern aligns with observations from Lake Kivu, where *Pisidium kenianum* and *Pisidium* cf. *viridarium* were restricted to streams, while *S.* cf*. hartmanni* was found in the lake proper [14]. Furthermore, we record no unionid species in the sampled volcanic barrier lakes, which aligns with the previous observations in these lakes, specifically lakes Bunyonyi, Mutanda, and Ruhondo [26,32]. It is worth noting that in the current study, the taxonomic challenges of certain groups, particularly bivalves, may have underestimated mollusc species richness in the volcanic barrier lakes region. Further rigorous taxonomic studies are recommended to refine these estimates.

### Ecological factors driving the snail distribution

Abiotic factors also play a crucial role in shaping the community composition and dynamics of aquatic macroinvertebrates, including molluscs [50–52]. For instance, lake morphology, particularly surface area, strongly correlates with macroinvertebrate richness [53]. The morphology influences the habitat availability and heterogeneity, which indirectly influence mollusc species composition and distribution. Lake Bunyonyi, with a surface area of approximately 56 km² and an amoeboid shape featuring numerous islands, provides diverse microhabitats and ecological niches, which may contribute to the relatively high number of mollusc species recorded there.

Physico-chemical parameters significantly influence snail distribution patterns on both regional and local scales in Africa [25]. Total dissolved solids (TDS), a surrogate for salinity and electrical conductivity (EC), strongly affect snail distributions in inland waterbodies [25,54]. We demonstrate a significant association of the EC with variation in the mollusc community composition in the selected five volcanic barrier lakes (p = 0.043, Fig 4), consistent with observations from other African regions, such as Niger and South Africa [25,54,55].

Similarly, extreme pH conditions have been shown to affect mollusc assemblages in freshwater systems [56]. The current study reports pH as a significant factor associated with mollusc community composition (p = 0.002, Fig 4), likely due to its impact on shell formation physiology via calcium ion leaching under acidic conditions [56]. Additionally, the presence of fishing activity is significantly associated with mollusc species composition in these lakes (p = 0.003, Fig 4, S8 Fig). These findings highlight the need for integrated approaches that consider both biotic and abiotic factors to manage and conserve mollusc biodiversity in these ecosystems.

The Albertine Rift volcanic barrier lakes experienced the intentional introduction of the North American crayfish (*P. clarkii*) in the 1960s, aimed at economic benefits without adequate consideration of ecological consequences [18]. *Procambarus clarkii* is known to drastically reduce populations of invertebrates (particularly molluscs), macrophytes, fish, and amphibians in aquatic systems. The ongoing invasion of *P. clarkii* in the sampled volcanic barrier lakes is evident (Fig 3) and has potentially influenced mollusc community composition (S8 Fig). However, current assessments are based solely on presence/absence data. We emphasise the need for comprehensive regional studies to quantify the ecological impacts of *P. clarkii*, particularly its effects on mollusc assemblages and broader aquatic biota.

In addition to *P. clarkii*, emerging invaders such as the gastropods *P. acuta* and the invasive Asian *M. tuberculata* lineage were detected (Fig 3). Invasive freshwater molluscs exert both direct and indirect pressures on native species by outcompeting and displacing them through resource monopolisation, habitat alteration, and modification of abiotic conditions [57]. These changes can facilitate the spread of novel diseases and contribute to ecosystem destabilisation [9,58]. The ecological consequences of such invasions can be severe, leading to declines in native biodiversity and disruptions in ecosystem functioning [9]. However, their impact varies depending on abiotic factors, biotic resistance, genetic diversity, and stochastic events [59]. These invasive species pose potential threats to mollusc diversity through native species replacement and potential community homogenisation, a phenomenon previously observed in Lake Malawi due to *M. tuberculata* [6,10]. The arrival of new global invaders in the region, such as *Pseudosuccinea columella* (Say, 1817) and *Pomacea canaliculata* (Lamarck, 1822) in East African waters such as Lake Naivasha, adds another layer of complexity to the problem [15]. Although in a broader context, currently invasive molluscs are not perceived as a serious threat to the African malacofauna, it should be noted that similar invasions have caused significant impacts in other regions [7]. Continuous monitoring is required to quantify the impact of these invasives on the malacological fauna of this region to better inform conservation efforts.

### Historical changes in molluscan fauna

Most ecosystems are currently undergoing community homogenisation driven by species and subspecies loss, making ecosystems more uniform [60]. This phenomenon has become a focal point in evolutionary and biodiversity conservation studies. Comparisons between historical records and recent surveys can provide useful indications of faunal change, but in the present case, they must be interpreted cautiously because of taxonomic uncertainty and differences in sampling design.

Our findings add to growing evidence for a creeping ecosystem homogenisation in the region, as several molluscan species and subspecies previously described by Mandahl-Barth [32] are becoming increasingly rare. Notable examples include *Bulinus ugandae*, *Gyraulus costulatus exilis*, and *Burnupia kempi* (Table 1). Conversely, a *Bulinus* morphologically resembling (*Bulinus* cf. *mutandaensis*) was found to be extremely rare in the lake proper, with very few specimens collected from the lake and more specimens collected from only one site in River Mucha, which drains Lake Mulehe into a swamp connected to Lake Mutanda. Phylogenetic analysis (S1 Fig) supports Brown [25]’s assertion that *B. mutandaensis* is a lacustrine form of *B. truncatus*, rather than a subspecies of *B. tropicus* as proposed by Mandahl-Barth [32].

Other interesting species previously described from the region are limpet-like species such as *Ferrissia toroensis* Mandahl-Barth, 1954, a critically endangered species and endemic to this region [25,61] and *Pettancylus ruandensis* Thiele, 1911. It remains unknown which species was found in Lake Bunyonyi (single specimen, C. Albrecht, pers. obs. 2013).

We also obtained new records of species not previously encountered in this lake system, including *M. tuberculata* and several Sphaeriidae species (Table 1). These species may have been overlooked in earlier, less exhaustive surveys, which primarily focused on the littoral zone. Furthermore, some might be ‘newcomers’, such as the invasive *M. tuberculata* (Asian lineage), which we recorded in Lake Mulehe.

Overall, the observed differences in molluscan community composition should be interpreted with caution, as ecosystem and limnological drivers were not directly assessed in this study. However, both the present and previous studies report environmental and limnological changes in the region that may influence molluscan assemblages. In addition, taxonomic uncertainties in both the current and historical records may affect cross-lake comparisons, and species richness estimates may change following future taxonomic revisions using, for example, population-level genomic approaches. Nevertheless, the main patterns reported here were consistent, suggesting that the conclusions are robust to current levels of taxonomic uncertainty.

Macroinvertebrate invasions (e.g., crayfish, *P. acuta*, and *M. tuberculata*), water pollution from hotel construction and extensive agriculture in the lake buffer zones, habitat loss exacerbated by buffer zone encroachment, and fish introductions are all driven by anthropogenic activities (reviewed in S1 Table; Fig 1). While some of the mollusc community patterns undoubtedly reflect true ecological changes, the possibility of discrepancies in species records must also be considered. For example, certain taxa reported in earlier surveys (such as *Sphaerium nyanzae* in 1931) may well correspond to species such as *Sphaerium* sp. in 2024.

### Biogeographical affinities

The molluscan fauna recorded in the volcanic barrier lakes appears to show a predominantly Nilotic affinities and is largely composed of species with broad Afrotropical distributions (Table 2). Notable examples include *B. truncatus*, which is widespread across Africa, including North Africa and the Congo Basin [14,25,41], and *R. natalensis*, which occurs widely in the Nile Basin and many other parts of Africa, including the Zaire Basin [25]. Similarly, *B. pfeifferi* is widely distributed in tropical Africa [25,33], while *E*. *keniana* are widely found in East Africa [33,34,45], with the latter extending its range from Ethiopia to Zambia [34,45]. Another widely distributed species is *B. tropicus*, ranging across East Africa from Ethiopia to South Africa [25,33]. *Pila ovata* is distributed from Egypt across much of Africa, including regions with Palearctic affinities and the Zaire Basin [25]. Additionally, *M. tuberculata* is commonly found in African waterbodies, including the Zaire Basin [25]; however, an invasive Asian lineage of this species was notably encountered in Lake Mulehe. *Segmentorbis* cf. *angustus* is likewise consistent with a broadly East and Central African distribution; related records assigned to *S*. *angustus* extend from Sudan to Natal and from Ethiopia to Lower Zaire [33]. It is found in major African great lakes such as Lake Malawi, Lake Tanganyika, Lake Turkana, Lake Victoria, and Lake Albert.

The introduction of alien and invasive species is also evident in these systems. *Physella acuta*, a widely invasive species, is particularly encountered in the Akagera-Victoria Nile system, i.e., Ruhondo and Burera systems, where it has been recorded in lakes such as Naivasha and Kivu tributaries [14,15], but it remains absent from the Edward Nile Basin, i.e., Bunyonyi, Mutanda and Mulehe system currently. Similarly, *Bulinus* cf*. mutandaensis,* a vulnerable species previously regarded as restricted to Lake Mutanda [25], was also for the first time recorded in Lake Mulehe. However, its precise taxonomic status relative to *Bulinus truncatus* remains unresolved and cannot be determined conclusively from the present data alone.

Geographically, there are limited differences in species composition between the Edward Nile Basin, which includes lakes Mutanda, Bunyonyi, and Mulehe in Uganda, and the Kagera-Victoria Nile system, represented by lakes Ruhondo and Burera on the Rwanda side (S4 Fig). For example, *B.* cf. *unicolor* was found only in the Akagera-Victoria Nile system and was absent from the Edward Nile Basin. Additionally, the invasive *P. acuta* was recorded in the Akagera-Victoria Nile system but was not detected in the Edward Nile system.

These findings underscore the complex biogeographic patterns in the volcanic barrier lakes, highlighting the interplay between native species, of which the majority have broad Afrotropical distributions, and invasive species. Together, these observations suggest that the volcanic barrier lakes form part of a broader East African freshwater fauna, while also showing limited local differentiation and emerging signatures of biological invasion.

## Conclusions

This study provides a recurrent assessment of the molluscan fauna of five volcanic barrier lakes in Uganda and Rwanda and shows that these understudied systems support a noteworthy freshwater mollusc diversity. The differences observed in mollusc community composition between historical records and the present survey suggest potential shifts in the assemblages of these lake ecosystems. However, these patterns should be interpreted with caution, as comparisons are constrained by uncertainties associated with historical taxonomic identifications and differences in sampling approaches. Some of the apparent changes may therefore reflect improvements in taxonomic resolution, particularly with the use of DNA barcoding in the present study.

Nevertheless, the presence of invasive and widely distributed species such as *Procambarus clarkii*, *Physella acuta*, and the Asian lineage of *Melanoides tuberculata* coincides with patterns consistent with biotic homogenisation. However, the extent to which these species contribute to changes in native assemblages remains uncertain. These invasions, coupled with habitat degradation caused by agricultural activities, pollution, and infrastructure development within lake riparian zones, pose threats to mollusc biodiversity.

Improving regional reference databases and applying emerging molecular tools, such as environmental DNA approaches, will be important for resolving taxonomic uncertainties and for more accurately documenting the conservation status of freshwater molluscs. There is an urgent need for the protection and sustainable management of these fragile ecosystems by international, national, and local authorities to mitigate further biodiversity loss.

## Supporting information

S1 Fig Phylogenetic evidence of a genetic relationship of *Bulinus* cf. *mutandaensis* within the *Bulinus tropicus*/*truncatus* complex. (DOCX)

S2 Fig A phylogenetic analysis to determine the invasive status of *Melanoides* species in the sampled lakes. (DOCX)

S3 Fig Maximum likelihood (ML) phylogenetic analysis to resolve the taxonomic discrepancies in the sampled *Biomphalaria* specimens. (DOCX)

S4 Fig Bray-Curtis similarity cluster analysis for mollusc faunal similarity. (DOCX)

S5 Fig Correlation matrix showing pairwise correlation among the environmental variable in Lake Bunyonyi, Mutanda, Mulehe, Ruhondo and Burera. (DOCX)

S6 Fig Haplotype network of *Euglesa* spp. based on 16S partial sequences. (PDF)

S7 Fig Rarefaction curve demonstrating the sampling completeness. (DOCX)

S8 Fig NMDS ordination of mollusc community composition showing the effects of binary habitat and disturbance variables (Yes/No). Points represent sampling sites, and ellipses indicate group dispersion.
(DOCX)

S1 Table A review of the historical ecosystem and limnology shifts that happened in lakes Mutanda, Mulehe, Bunyonyi, Ruhondo, and Burera over the last 100 yrs. (DOCX)

S2 Table Results of BLAST searches, indicating species, with voucher code, the associated BLAST hits with % similarity (in brackets), NCBI GenBank accession numbers and the countries of origin. (DOCX)

S3 Table Characteristics of the sampled lakes and streams, including altitude, physical and chemical characteristics of the water, substrate type, utilisation and the degree of anthropogenic disturbance. (DOCX)

S4 Table Snail abundance raw data. Where: Lake Ruhondo sites; RW01, RW02, RW03, RW04, RW05, RW06, RW07, RW08, RW09, RW10, RW11, RW12, RW13, RW14, RW15, RW16, RW17, RW18, RW19, RWA20, RW21. Lake Burera sites; RW22, RW23, RW24, RW25, RW26, RW27. Lake Bunyonyi; UG01, UG04, UG08, UG09, UG16. Stream to Lake Bunyonyi; UG02, UG17. Lake Mutanda; UG03, UG05, UG14, UG15. R. Mucha drains L. Mulehe; UG06. L. Mulehe; UG07, UG10, UG11, UG12, UG13.
(DOCX)

S5 Table 1) NMDS score values for mollusc community data, 2) NMDS score values for environmental variables. (DOCX)

S1 Data sheet Environmental variable raw data. Where: Lake Ruhondo sites; RWA13, RWA14, RWA15, RWA17, RWA18, RWA19, RWA20, RWA21, RWA22, RWA23, RWA24, RWA25, Lake Burera sites; RWA08. Lake Bunyonyi; UGA04, UGA07, UGA08, UGA09. Mutanda-Mulehe System sites; UGA14, UGA15, UGA10, UGA13, UGA08.
(XLSX)

S2 Data sheet Mollusc community raw data. Where: Lake Ruhondo sites; RWA13, RWA14, RWA15, RWA17, RWA18, RWA19, RWA20, RWA21, RWA22, RWA23, RWA24, RWA25, Lake Burera sites; RWA08. Lake Bunyonyi; UGA04, UGA07, UGA08, UGA09. Mutanda-Mulehe System sites; UGA14, UGA15, UGA10, UGA13, UGA08.
(XLSX)

## References

[1] Darwall W, Smith K, Lowe T, Vié JC. The status and distribution of freshwater biodiversity in Eastern Africa. IUCN SSC Freshw Biodivers Assess Program. 2005. p. 1–36.

[2] De Crop W, Verschuren D. Mixing regimes in the equatorial crater lakes of western Uganda. Limnologica. 2021;90:125891. doi: 10.1016/j.limno.2021.125891

[3] Beadle LC. The inland waters of tropical Africa: an introduction to tropical limnology. 2nd ed. New York: Longman Group Limited; 1981. 267–9 p.

[4] Dumont HJ. The Nile: origin, environments, limnology and human use. Vol. 89. Springer Science & Business Media; 2009. 263–9 p.

[5] Stager JC, Day JJ, Santini S. Comment on “Origin of the superflock of cichlid fishes from Lake Victoria, East Africa”. Science. 2004;304(5673):325–9. doi: 10.1126/science.1091978 15143263

[6] Van Bocxlaer B, Albrecht C. Ecosystem change and establishment of an invasive snail alter gastropod communities in long-lived Lake Malawi. Hydrobiologia. 2014;744(1):307–16. doi: 10.1007/s10750-014-2093-0

[7] Graf D. Freshwater molluscs of Africa: diversity, distribution, and conservation. Gland: IUCN. 2011.

[8] Albrecht C, Kipyegon JK, Junginger A, Clewing C. Returners and New Arrivals After the Crash: Intermediate Hosts and Global Invaders Dominate Gastropod Fauna of Lake Naivasha, Kenya. Diversity. 2025;17(4):265. doi: 10.3390/d17040265

[9] Lopes-Lima M, Lopes-Lima A, Burlakova L, Douda K, Alonso Á, Karatayev A, et al. Non-native freshwater molluscs: a brief global review of species, pathways, impacts and management strategies. Hydrobiologia. 2025;852(5):1005–28. doi: 10.1007/s10750-024-05780-3

[10] Van Bocxlaer B, Clewing C, Mongindo Etimosundja J-P, Kankonda A, Wembo Ndeo O, Albrecht C. Recurrent camouflaged invasions and dispersal of an Asian freshwater gastropod in tropical Africa. BMC Evol Biol. 2015;15:1–18. doi: 10.1186/s12862-015-0296-2 25886047 PMC4373078

[11] Genner MJ, Michel E, Erpenbeck D, De Voogd N, Witte F, Pointier J-P. Camouflaged invasion of Lake Malawi by an Oriental gastropod. Mol Ecol. 2004;13(8):2135–41. doi: 10.1111/j.1365-294X.2004.02222.x 15245389

[12] Rahel FJ. Homogenisation of freshwater faunas. Annu Rev Ecol Syst. 2002;33(1):291–315.

[13] Appleton CC. Alien and invasive fresh water Gastropoda in South Africa. African J Aquat Sci. 2003;28(1):69–81. doi: 10.2989/16085914.2003.9626602

[14] Dusabe MC, Kalinda C, Clewing C, Hyangya BL, Van Bocxlaer B, Albrecht C. Environmental perturbations and anthropogenic disturbances determine mollusc biodiversity of Africa’s explosive Lake Kivu. J Great Lakes Res. 2024;50(3):102339. doi: 10.1016/j.jglr.2024.102339

[15] Albrecht C, Clewing C, Seebens H, Chibwana FD, Da Silva EL, Leal MF, et al. When one global invasion hides another—cryptic interspecific invasion in freshwater gastropods. Divers Distrib. 2025;31(1):e13958.

[16] Magumba MK. Physical, chemical, algal composition and primary production in the four Kisoro minor lakes. Jinja, Uganda: Fisheries Resources Research Institute; 2000.

[17] Twardochleb LA, Olden JD, Larson ER. A global meta-analysis of the ecological impacts of nonnative crayfish. Freshw Sci. 2013;32(4):1367–82.

[18] Madzivanzira TC, South J, Wood LE, Nunes AL, Weyl OLF. A review of freshwater crayfish introductions in Africa. Rev Fish Sci Aquac. 2020;29(2):218–41.

[19] Hofkin BV, Koech DK, Oumaj J, Loker ES. The North American crayfish Procambarus clarkii and the biological control of schistosome-transmitting snails in Kenya: Laboratory and field investigations. Biol Control. 1991;1(3):183–7. doi: 10.1016/1049-9644(91)90065-8 1928568

[20] Mkoji GM, Hofkin BV, Kuris AM, Stewart-Oaten A, Mungai BN, Kihara JH, et al. Impact of the crayfish Procambarus clarkii on Schistosoma haematobium transmission in Kenya. Am J Trop Med Hyg. 1999;61(5):751–9. doi: 10.4269/ajtmh.1999.61.751 10586907

[21] Khalil MT, Sleem SH. Can the freshwater crayfish eradicate schistosomiasis in Egypt and Africa. J Am Sci. 2011;7(7):457–62.

[22] Foster J, Harper D. Status and ecosystem interactions of the invasive Louisianan red swamp crayfish Procambarus clarkii in East Africa. In: Gherardi F, editor Biological invaders in inland waters: Profiles, distribution, and threats. Dordrecht: Springer; 2007. p. 91–101.

[23] Mandahl-Barth G. Key to the identification of east and central African freshwater snails of medical and veterinary importance. Bull World Health Organ. 1962;27(1):135–50. 14469160 PMC2555811

[24] Brown DS, Wright CA. On a polyploid complex of freshwater snails (Planorbidae: Bulinus) in Ethiopia. J Zool. 1972;167(1):97–132.

[25] Brown D. Freshwater Snails of Africa and their Medical Importance. 2nd ed. UK: Taylor & Francis; 1994. 584 p.

[26] Thiele J. Mollusken der deutschen Zentral-Afrika-Expedition. Wissenschaftliche Ergebnisse der deutschen Zentral-Afrika-Expedition, 1907–1908. Leipzig: Klinkhardt & Biermann; 1911. p. 175–214.

[27] Worthington EB. A report on the fisheries of Uganda investigated by the Cambridge expedition to the East African lakes, 1930-31 with 3 appendices, 5 maps and 21 other illustrations. Landon: Zoological laboratory; 1932.

[28] Habimana V, Nsabimana A. Water Physico-Chemical Characteristics of the Lakes Burera and Ruhondo, Rwanda. Rwanda J Eng Sci Technol Environ. 2020;3(2). doi: 10.4314/rjeste.v3i2.5

[29] Papius DMT, William O, Alex B, Dismas M, Jimmy O, Vincent K. Status of Kigezi minor lakes: A limnological survey in the lakes of Kisoro, Kabale and Rukungiri districts. Int J Water Resour Environ Eng. 2016;8(5):60–73.

[30] Habiyakare T, Zhou N. Water Resources Conflict Management of Nyabarongo River and Kagera River Watershed in Africa. J Water Resour Prot. 2015;07(12):889–96. doi: 10.4236/jwarp.2015.712073

[31] Nzabuheraheza FD, Nyiramugwera AN. Food security status in developing countries: a case study of Burera and Musanze districts of Rwanda. African J Food Agric Nutr Dev. 2017;17(3):12413–26.

[32] Mandahl-Barth G. The freshwater molluscs of Uganda and adjacent territories. Koninklijk Museum van Belgisch-Congo; 1954. 182–5 p.

[33] Danish Bilharziasis Laboratory. A field guide to African freshwater snails. 2nd ed. World Health Organization; 1987.

[34] Mandahl-Barth G. Studies on African freshwater bivalves. Danish bilharziasis laboratory; 1988.

[35] Wilke T, Davis GM, Qiu D, Spear RC. Extreme mitochondrial sequence diversity in the intermediate schistosomiasis host Oncomelania hupensis robertsoni: another case of ancestral polymorphism? Malacologia. 2006;48(1/2):143.

[36] Wilke T, Davis GM. Infraspecific mitochondrial sequence diversity in Hydrobia ulvae and Hydrobia ventrosa (Hydrobiidae: Rissooidea: Gastropoda): do their different life histories affect biogeographic patterns and gene flow? Biol J Linn Soc. 2000;70(1):89–105.

[37] Palumbi SR, Martin A, Romano S, McMillan WO, Stice L, Grabowski G. The Simple Fool’s Guide to PCR. Honolulu: University of Hawaii; 1991.

[38] Hall TA. BioEdit: a user-friendly biological sequence alignment editor and analysis program for Windows 95/98/NT. Nucleic acids symposium series. Oxford; 1999. p. 95–8.

[39] Leigh JW, Bryant D. popart: full‐feature software for haplotype network construction. Methods Ecol Evol. 2015;6(9):1110–6. doi: 10.1111/2041-210x.12410

[40] Jørgensen A, Kristensen TK, Madsen H. A molecular phylogeny of apple snails (Gastropoda, Caenogastropoda, Ampullariidae) with an emphasis on African species. Zool Scr. 2008;37(3):245–52.

[41] Tumwebaze I, Clewing C, Dusabe MC, Tumusiime J, Kagoro-Rugunda G, Hammoud C, et al. Molecular identification of Bulinus spp. intermediate host snails of Schistosoma spp. in crater lakes of western Uganda with implications for the transmission of the Schistosoma haematobium group parasites. Parasit Vectors. 2019;12(1):565. doi: 10.1186/s13071-019-3811-2 31775865 PMC6882369

[42] Mahulu A, Clewing C, Stelbrink B, Chibwana FD, Tumwebaze I, Russell Stothard J, et al. Cryptic intermediate snail host of the liver fluke Fasciola hepatica in Africa. Parasit Vectors. 2019;12(1):573. doi: 10.1186/s13071-019-3825-9 31801595 PMC6894237

[43] Schultheiß R, Van Bocxlaer B, Riedel F, von Rintelen T, Albrecht C. Disjunct distributions of freshwater snails testify to a central role of the Congo system in shaping biogeographical patterns in Africa. BMC Evol Biol. 2014;14(1):42. doi: 10.1186/1471-2148-14-42 24597925 PMC4015641

[44] Gu QH, Husemann M, Wu HH, Dong J, Zhou CJ, Wang XF, et al. Phylogeography of Bellamya (Mollusca: Gastropoda: Viviparidae) snails on different continents: contrasting patterns of diversification in China and East Africa. BMC Evol Biol. 2019;19(1):82. doi: 10.1186/s12862-019-1397-0 30898091 PMC6429760

[45] Clewing C, Geertz T, Rassam H, Woldekiros TH, Albrecht C. Freshwater diversity at a biogeographic edge zone: the high-mountain pea-clams of Ethiopia. Syst Biodivers. 2022;20(1):1–15.36970113

[46] Van Damme D, Van Bocxlaer B. Freshwater molluscs of the Nile Basin, past and present. In: Dumont HJ, editor. The Nile. Monographiae Biologicae, vol 89. Dordrecht: Springer; 2009. p. 585–629.

[47] Holmberg R, Madsen H, Kristensen T, Jørgensen A. Gastropod distribution in Lakes George and Edward, Uganda, relative to copper and cobalt levels. Afr J Aquat Sci. 2011;36(2):191–6. doi: 10.2989/16085914.2011.589117

[48] Besser JM, Dorman RA, Hardesty DL, Ingersoll CG. Survival and Growth of Freshwater Pulmonate and Nonpulmonate Snails in 28-Day Exposures to Copper, Ammonia, and Pentachlorophenol. Arch Environ Contam Toxicol. 2016;70(2):321–31. doi: 10.1007/s00244-015-0255-3 26747374

[49] Miller JP, Ramos MA, Hauffe T, Delicado D. Global species richness of hydrobiid snails determined by climate and evolutionary history. Freshw Biol. 2018;63(10):1225–39.

[50] Hämäläinen H, Luotonen H, Koskenniemi E, Liljaniemi P. Inter-annual variation in macroinvertebrate communities in a shallow forest lake in eastern Finland during 1990–2001. Hydrobiologia. 2003;506–509(1–3):389–97. doi: 10.1023/b:hydr.0000008581.86095.0b

[51] Hauffe T, Albrecht C, Wilke T. Assembly processes of gastropod community change with horizontal and vertical zonation in ancient Lake Ohrid: a metacommunity speciation perspective. Biogeosciences. 2016;13(10):2901–11. doi: 10.5194/bg-13-2901-2016

[52] Chi S, Hu J, Zheng J, Li S, Li M, Hu J. The role of abiotic and biotic factors within influencing macroinvertebrate communities in a subtropical eutrophic reservoir with thermal stratification. Chem Ecol. 2024;40(5):535–52.

[53] Zenker A, Baier B. Relevance of abiotic criteria used in German lake typology for macroinvertebrate fauna. Hydrobiologia. 2009;636(1):379–92. doi: 10.1007/s10750-009-9967-6

[54] Marie MAS, El-Deeb FAA, Hasheesh WS, Mohamed RA, Sayed SSM. Impact of seasonal water quality and trophic levels on the distribution of various freshwater snails in four Egyptian governorates. Appl Ecol Environ Sci. 2015;3(4):117–26.

[55] Meier-Brook C, Haas D, Winter G, Zeller T. Hydrochemical factors limiting the distribution of Bulinus truncatus (Pulmonata: Planorbidae). Am Malacol Bull. 1987.

[56] Tchakonté S, Ajeagah GA, Diomandé D, Camara AI, Ngassam P. Diversity, dynamic and ecology of freshwater snails related to environmental factors in urban and suburban streams in Douala–Cameroon (Central Africa). Aquat Ecol. 2014;48:379–95.

[57] Preston DL, Crone ER, Miller‐ter Kuile A, Lewis CD, Sauer EL, Trovillion DC. Non‐native freshwater snails: a global synthesis of invasion status, mechanisms of introduction, and interactions with natural enemies. Freshw Biol. 2022;67(2):227–39.

[58] Geist J, Benedict A, Dobler AH, Hoess R, Hoos P. Functional interactions of non-native aquatic fauna with European freshwater bivalves: implications for management. Hydrobiologia. 2025;852(5):1397–419. doi: 10.1007/s10750-022-05121-2

[59] Sousa R, Nogueira JG, Padilha J. Moving from the species to the population level in biological invasions. Glob Chang Biol. 2024;30(7):e17396. doi: 10.1111/gcb.17396 38958102

[60] Brodersen J, Seehausen O. Why evolutionary biologists should get seriously involved in ecological monitoring and applied biodiversity assessment programs. Evol Appl. 2014;7(9):968–83. doi: 10.1111/eva.12215 25553061 PMC4231589

[61] Albrecht C, Clewing C. *Pettancylus toroensis*. The IUCN Red List of Threatened Species. 2022. e.T184630A187684642 p. [Accessed 2025 September 22]. Available from: https://www.iucnredlist.org/

