# Supplementary material for: Invaders taking over—Mollusc faunal change in volcanic barrier lakes of the Albertine Rift biodiversity hotspot
Source: PLoS One. 2026 Jun 30;21(6):e0352648. doi: 10.1371/journal.pone.0352648 (PMC13318018; doi:10.1371/journal.pone.0352648)
Supplement: S1 Fig — (DOCX) [file pone.0352648.s001.docx]

S2 **Fig.** A phylogenetic evidence of a genetic relationship of *Bulinus* cf. *mutandaensis* within the *Bulinus* *tropicus*/*truncatus* complex. (a) Phylogeography of *Bulinus* spp. A haplotype network showing the distribution of the haplotypes among lakes where UGSB 29948 represents *Bulinus* cf. *mutandaensis* haplotype. Haplotypes are coloured according to their geographical occurrence. (b) Maximum likelihood phylogenetic tree showing the genetic relationship between *B.* cf. *mutandaensis* with other *Bulinus* spp. Bootstrap support values above 0.50 are shown on the nodes. The scale bar represents the number of substitution per site according to the model of sequence evolution.

**
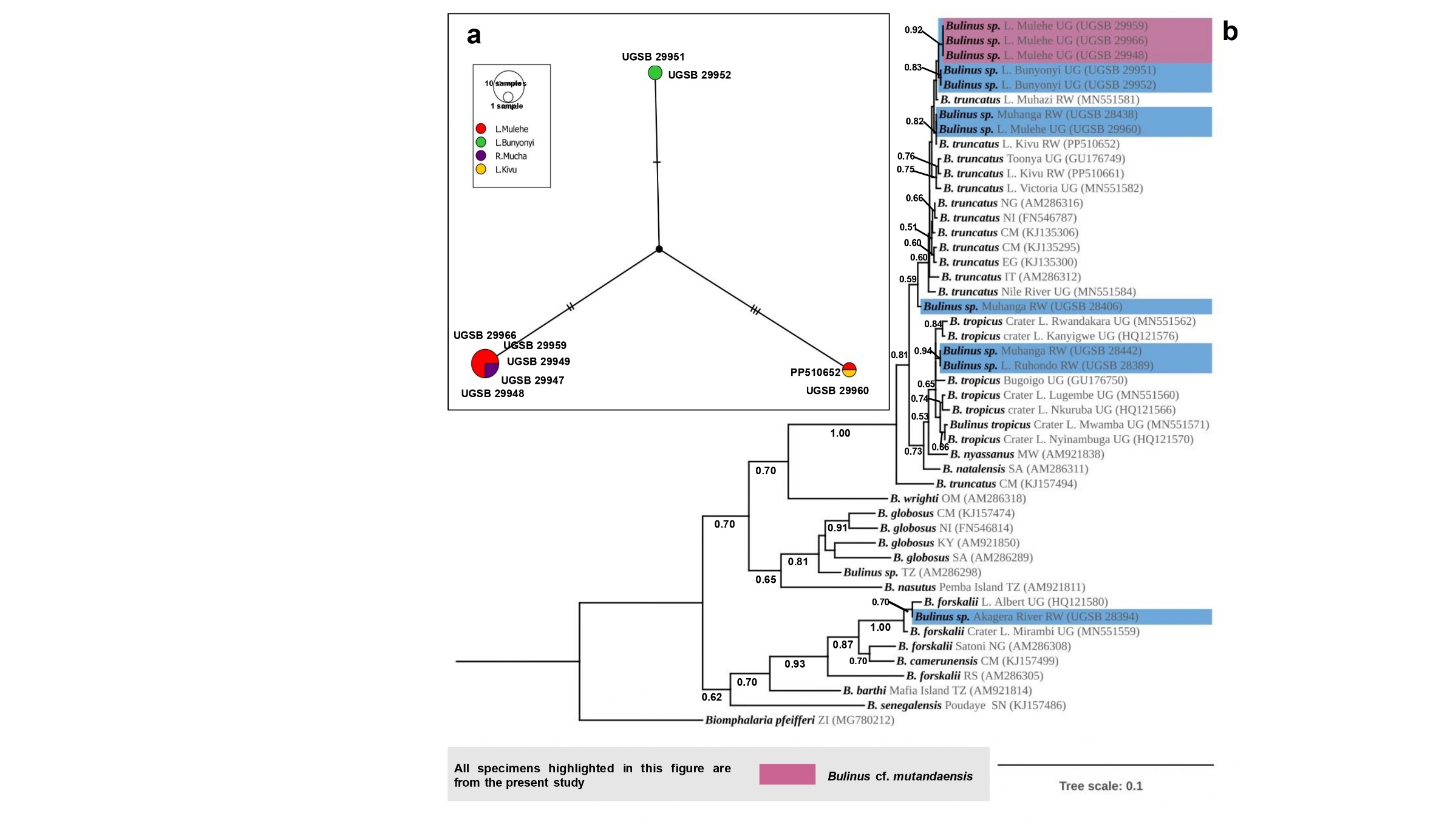
**
