# Supplementary material for: Invaders taking over—Mollusc faunal change in volcanic barrier lakes of the Albertine Rift biodiversity hotspot
Source: PLoS One. 2026 Jun 30;21(6):e0352648. doi: 10.1371/journal.pone.0352648 (PMC13318018; doi:10.1371/journal.pone.0352648)

S3 Fig. A phylogenetic analysis to determine the invasive status of Melanoides species in the sampled lakes. In this analysis, 72 sequences were adopted from Van Bocxlaer et al*.* (2015), analysed with four *Melanoides tuberculata* sequences from the present study. Orange colour represents Malawi-Congo invasion, grey Tanganyikan invasion, and blue specimen from the present study.


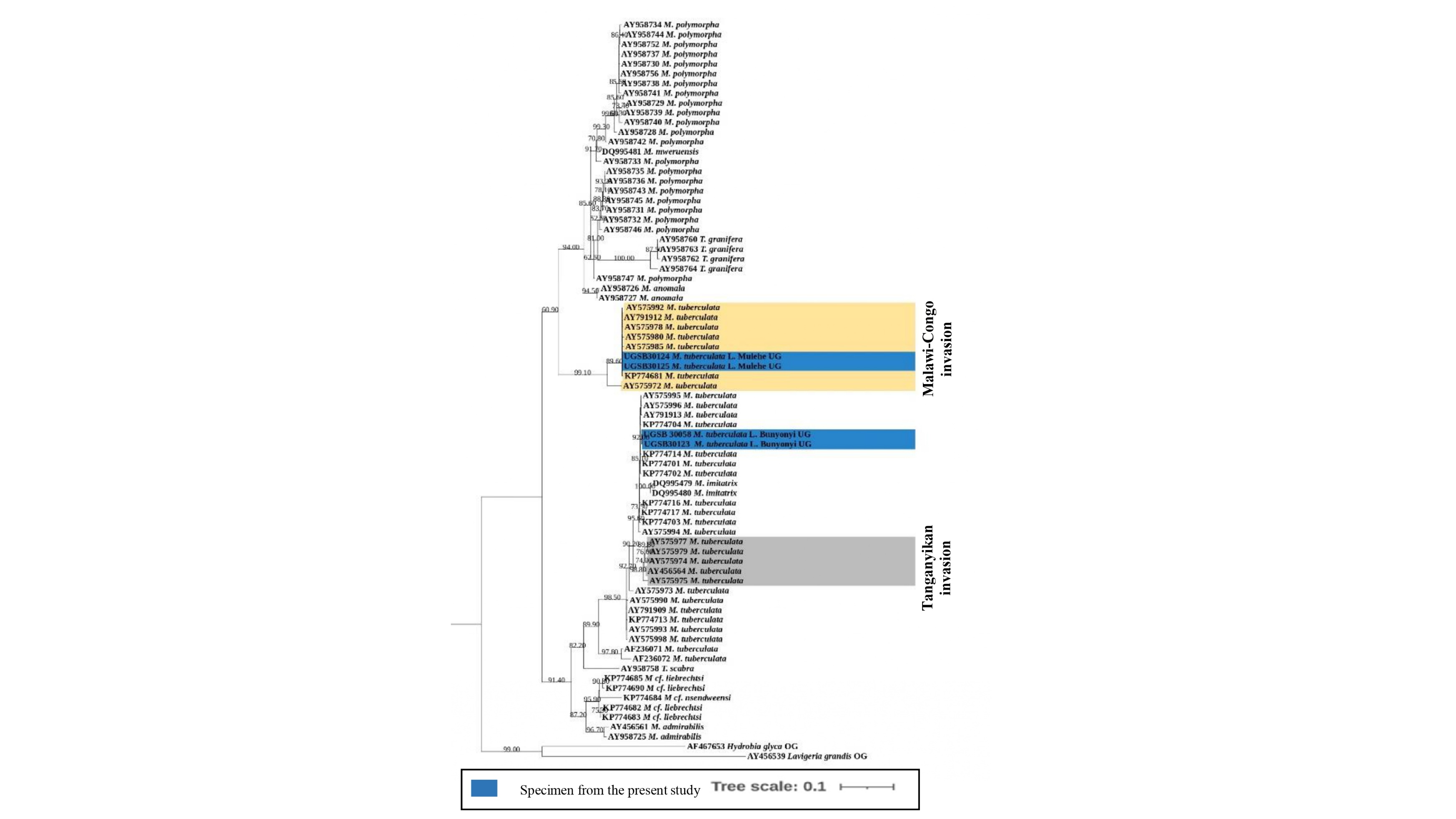

Supplement: S2 Fig — (DOCX) [file pone.0352648.s002.docx]
