# Supplementary material for: Invaders taking over—Mollusc faunal change in volcanic barrier lakes of the Albertine Rift biodiversity hotspot
Source: PLoS One. 2026 Jun 30;21(6):e0352648. doi: 10.1371/journal.pone.0352648 (PMC13318018; doi:10.1371/journal.pone.0352648)
Supplement: S3 Fig — (DOCX) [file pone.0352648.s003.docx]

S4 Fig. Maximum likelihood (ML) phylogenetic analysis to resolve the taxonomic discrepancies in the sampled *Biomphalaria* specimens. Where: DRC-Democratic Republic of Congo, KY-Kenya, MW-Malawi, RW-Rwanda, UG-Uganda representing the country of origin.

**
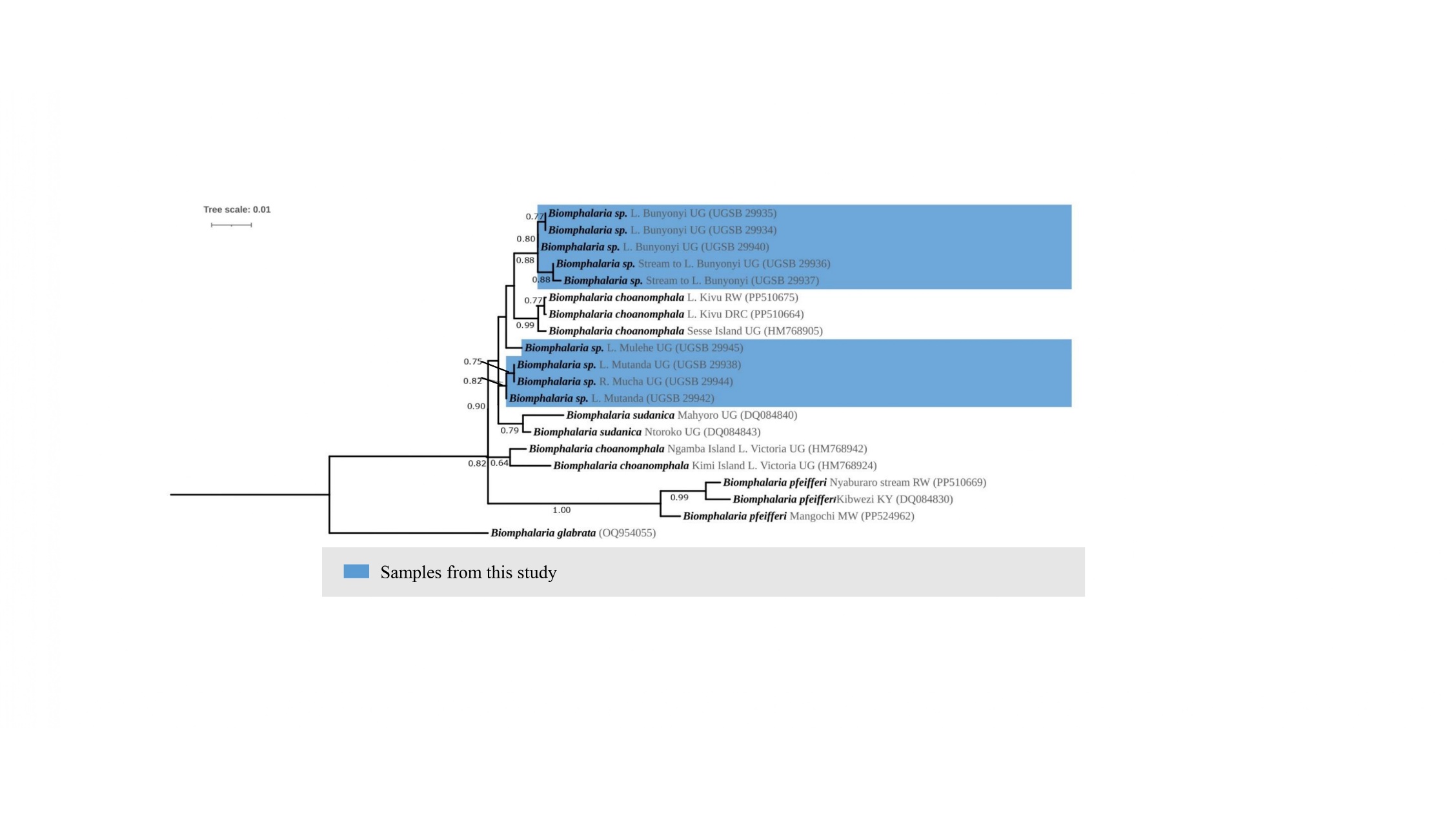
**
