# Supplementary material for: Invaders taking over—Mollusc faunal change in volcanic barrier lakes of the Albertine Rift biodiversity hotspot
Source: PLoS One. 2026 Jun 30;21(6):e0352648. doi: 10.1371/journal.pone.0352648 (PMC13318018; doi:10.1371/journal.pone.0352648)
Supplement: S4 Fig — (DOCX) [file pone.0352648.s004.docx]

S5 **Fig.** Bray-Curtis similarity cluster analysis for mollusc faunal similarity. Where: Lake Ruhondo sites; RW01, RW02, RW03, RW04, RW05, RW06, RW07, RW08, RW09, RW10, RW11, RW12, RW13, RW14, RW15, RW16, RW17, RW18, RW19, RWA20, RW21. Lake Burera sites; RW22, RW23, RW24, RW25, RW26, RW27. Lake Bunyonyi; UG01, UG04, UG08, UG09, UG16. Stream to Lake Bunyonyi; UG02, UG17. Lake Mutanda; UG03, UG05, UG14, UG15.  R. Mucha drains L. Mulehe; UG06.  L. Mulehe; UG07, UG10, UG11, UG12, UG13.

***
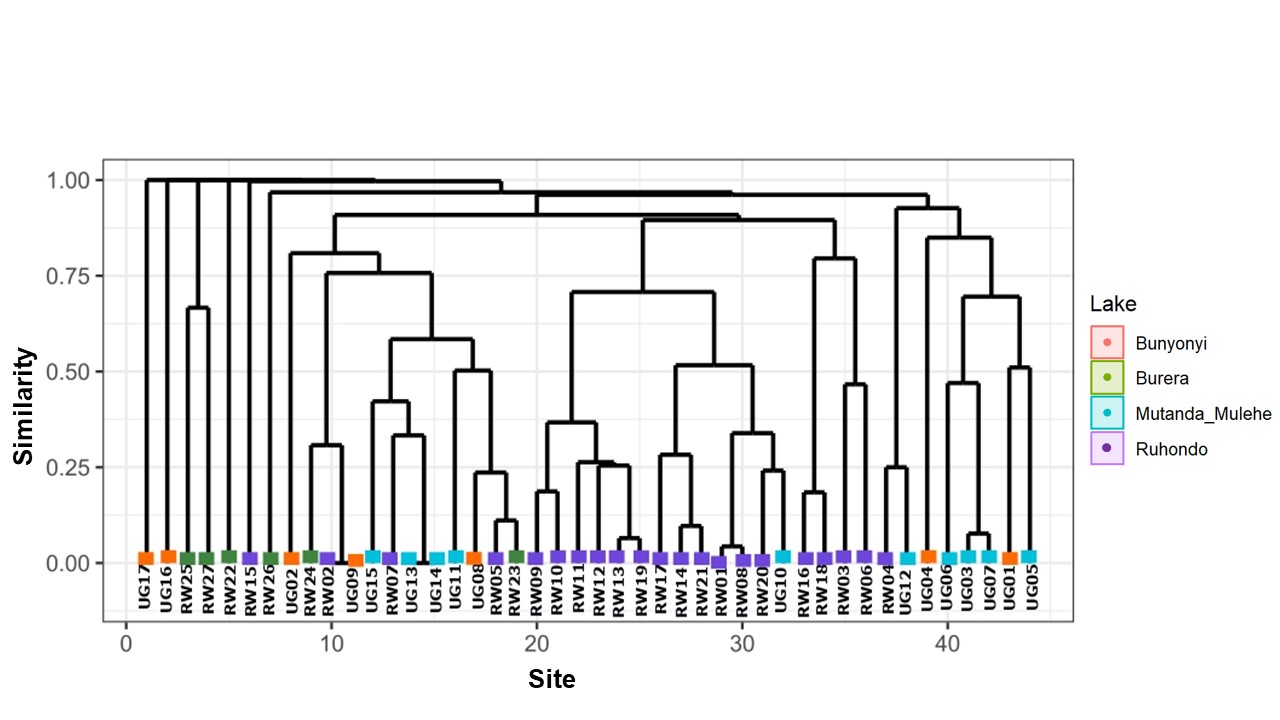
***
