# Supplementary material for: Invaders taking over—Mollusc faunal change in volcanic barrier lakes of the Albertine Rift biodiversity hotspot
Source: PLoS One. 2026 Jun 30;21(6):e0352648. doi: 10.1371/journal.pone.0352648 (PMC13318018; doi:10.1371/journal.pone.0352648)

**S9 Fig.** Correlation matrix showing pairwise correlation among the environmental variable in Lake Bunyonyi, Mutanda, Mulehe, Ruhondo and Burera.


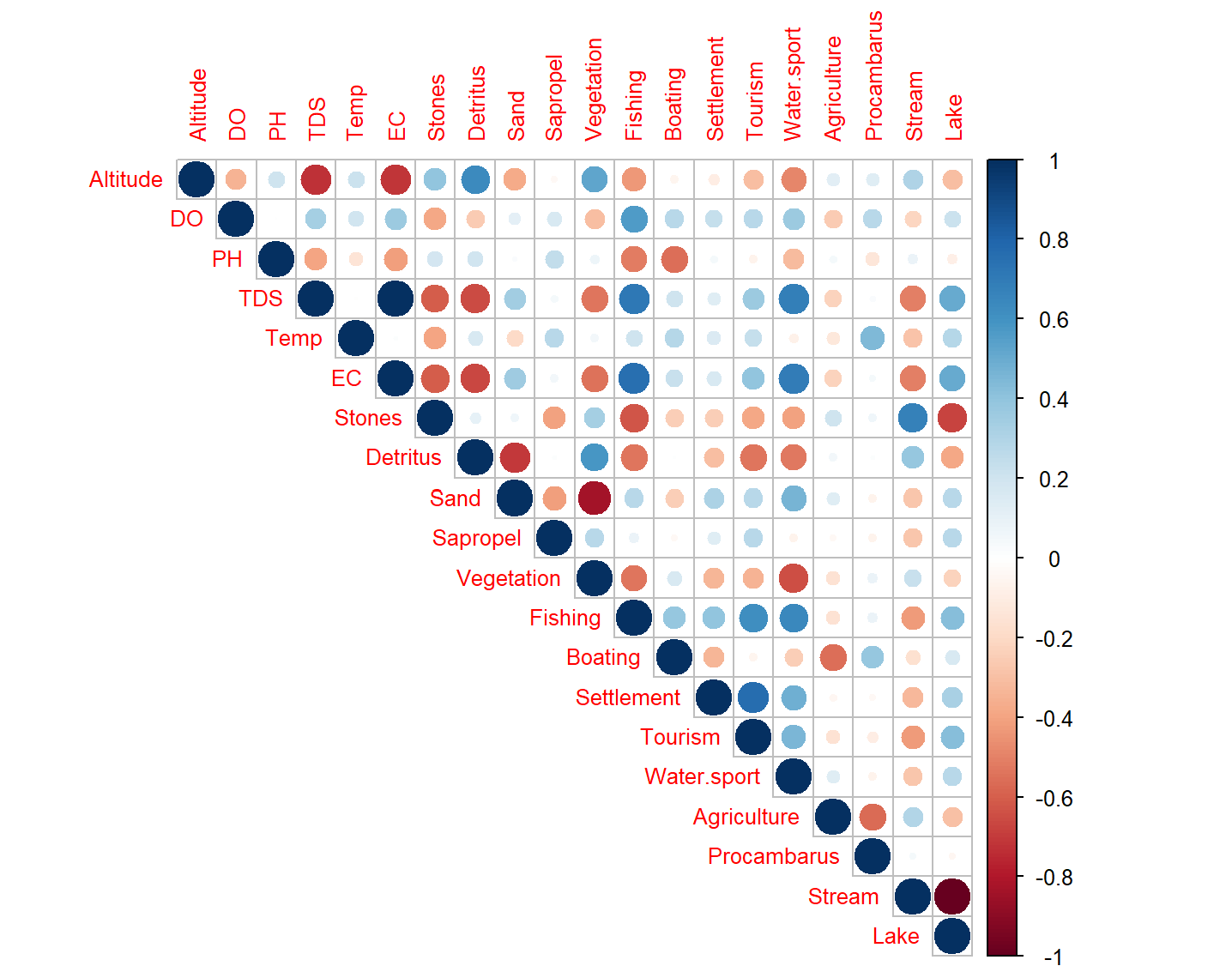

Supplement: S5 Fig — (DOCX) [file pone.0352648.s005.docx]
