# Supplementary material for: Invaders taking over—Mollusc faunal change in volcanic barrier lakes of the Albertine Rift biodiversity hotspot
Source: PLoS One. 2026 Jun 30;21(6):e0352648. doi: 10.1371/journal.pone.0352648 (PMC13318018; doi:10.1371/journal.pone.0352648)
Supplement: S6 Fig — (PDF) [file pone.0352648.s006.pdf]

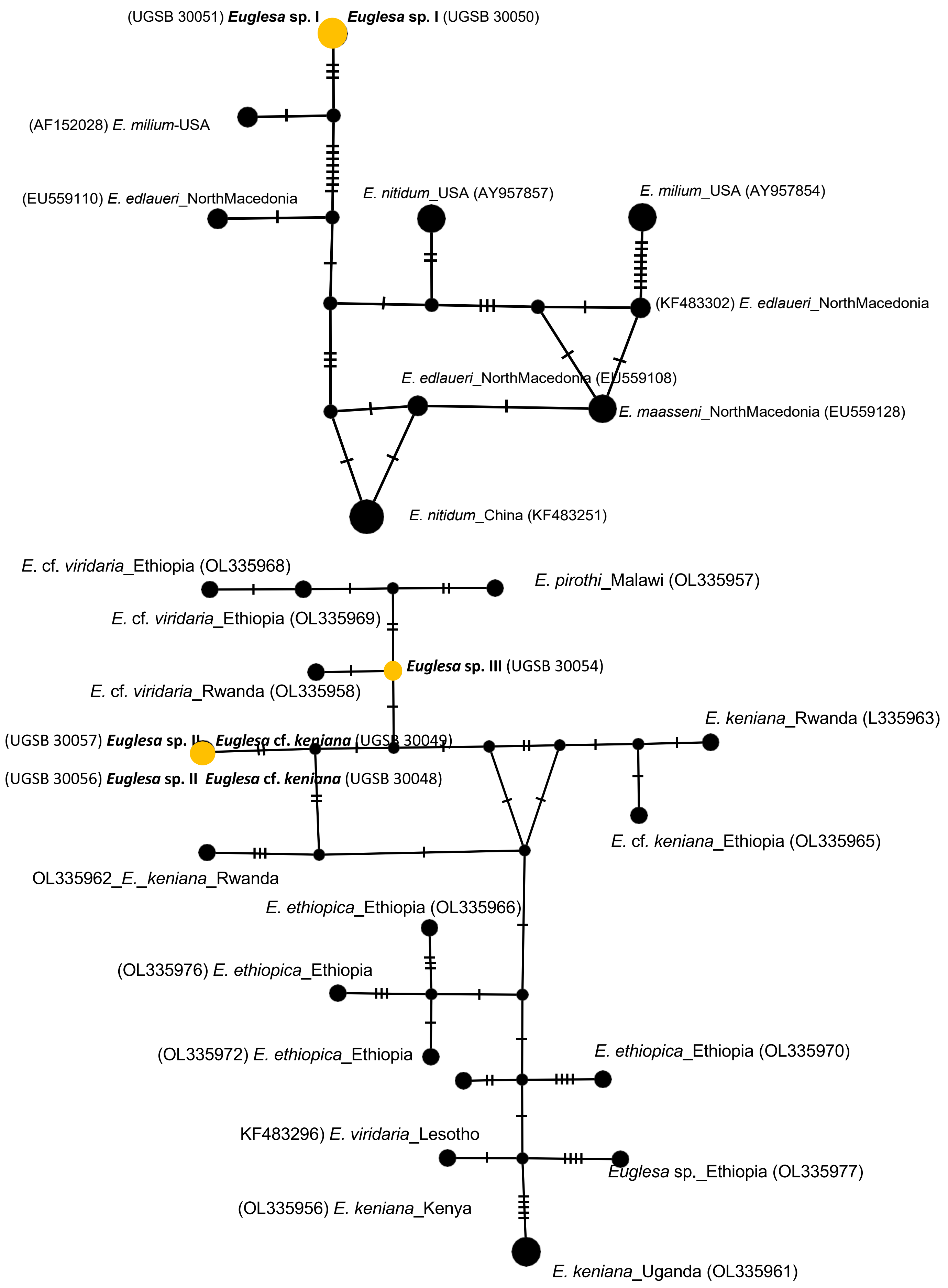

Fig S10. Haplotype network of *Euglesa* spp. based on 16S partial sequences. Where [yellow circle] represents specimens collected from Lake Bunyonyi in the present study
