# Supplementary material for: Invaders taking over—Mollusc faunal change in volcanic barrier lakes of the Albertine Rift biodiversity hotspot
Source: PLoS One. 2026 Jun 30;21(6):e0352648. doi: 10.1371/journal.pone.0352648 (PMC13318018; doi:10.1371/journal.pone.0352648)

**S14 Fig**. Rarefaction curve demonstrating the sampling completeness. They were constructed from species abundance data, with 95% confidence intervals estimated via a 50-times bootstrap resampling.


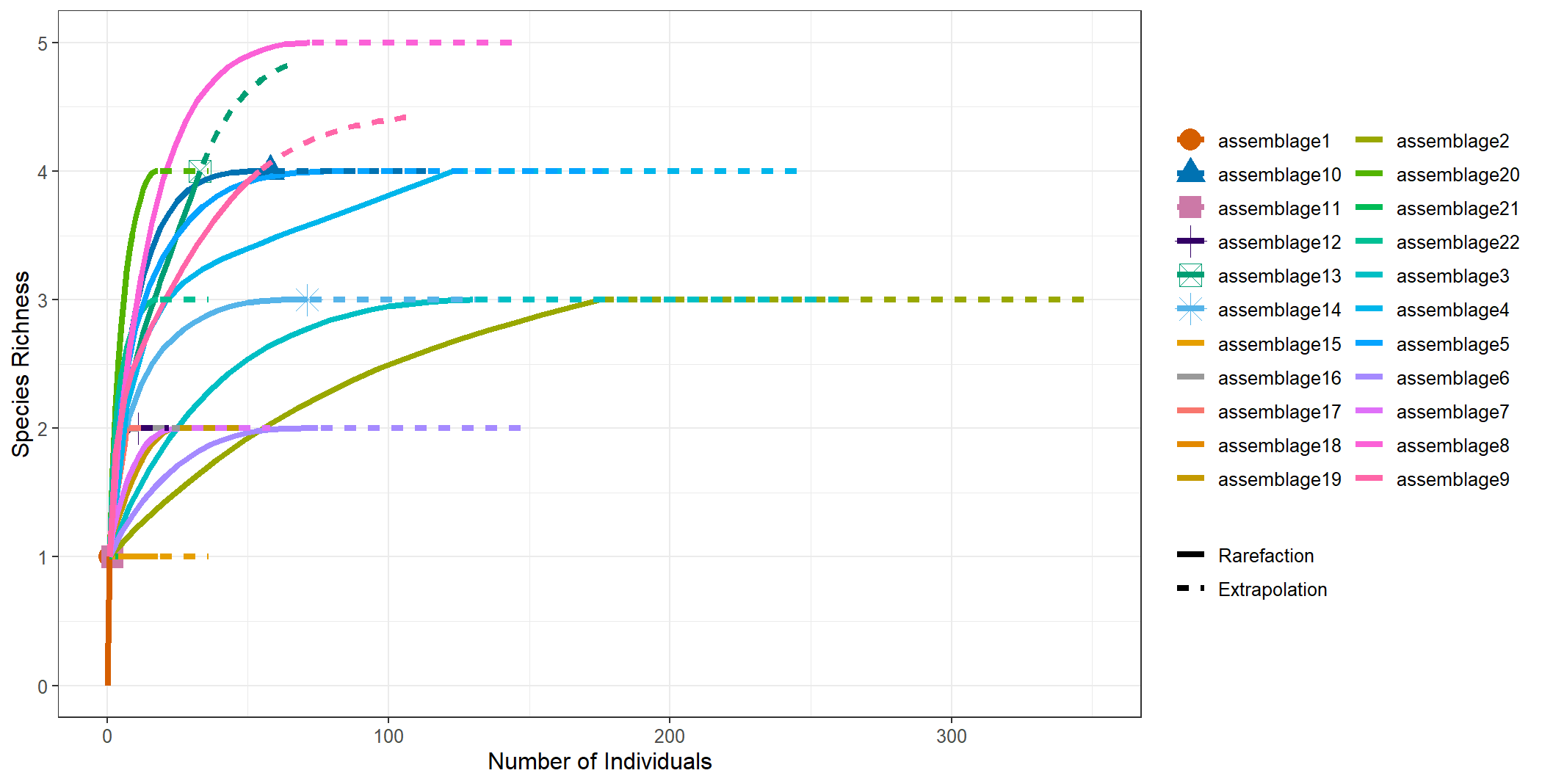

Supplement: S7 Fig — (DOCX) [file pone.0352648.s007.docx]
