# Supplementary material for: Invaders taking over—Mollusc faunal change in volcanic barrier lakes of the Albertine Rift biodiversity hotspot
Source: PLoS One. 2026 Jun 30;21(6):e0352648. doi: 10.1371/journal.pone.0352648 (PMC13318018; doi:10.1371/journal.pone.0352648)

**S15 Fig.** NMDS ordination of mollusc community composition showing the effects of binary habitat and disturbance variables (Yes/No). Points represent sampling sites, and ellipses indicate group dispersion.


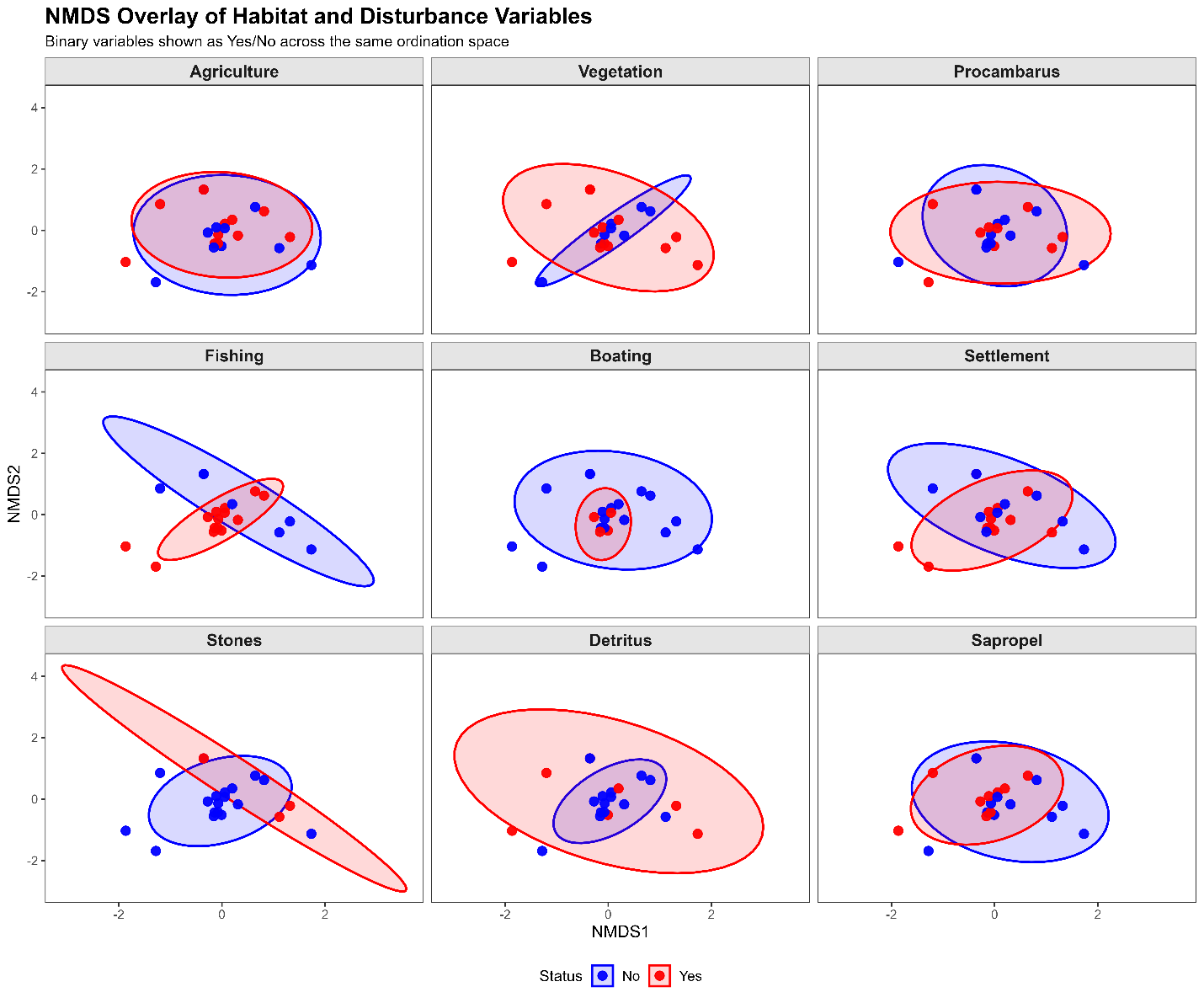

Supplement: S8 Fig — Points represent sampling sites, and ellipses indicate group dispersion. (DOCX) [file pone.0352648.s008.docx]
