# Supplementary material for: Invaders taking over—Mollusc faunal change in volcanic barrier lakes of the Albertine Rift biodiversity hotspot
Source: PLoS One. 2026 Jun 30;21(6):e0352648. doi: 10.1371/journal.pone.0352648 (PMC13318018; doi:10.1371/journal.pone.0352648)
Supplement: S1 Table — (DOCX) [file pone.0352648.s009.docx]

# Supporting information

## S1 Table. A review of the historical ecosystem and limnology shifts that happened in lakes Mutanda, Mulehe, Bunyonyi, Ruhondo, and Burera for over the last 100 yrs. Where: [1] (Green, 2009), [2] (Green, 1965), [3] (Kamanyi, Ndawula and Magumba, 2000), [4] (De Vos, Snoeks and van den Audenaerde, 1990), [5] (Isumbisho *et al.*, 2011), [6] (Madzivanzira et al., 2020), [7] (Saturday *et al.*, 2023), [8] (Magumba, 2000), [9] (Saturday, Kangume and Bamwerinde, 2023), [10] (Habimana and Nsabimana, 2020).

| **Ecological change** |  | **Timeframe** | | | |
| --- | --- | --- | --- | --- | --- |
|  | **1903-1923** | **1924-1944** | **1945-1965** | **1966-1986** | **≥1987** |
| **Phytoplankton change** | | | | | |
| **Bunyonyi** |  | In 1931, *Botryococcus* and a few diatoms  (*Synedra*) dominated [1] | In 1961, *Microcystis* and *Anabaenopsis*  dominated [1] |  | In 1990, *Microcystis* and *Ceratium* dominated [1] |
| **Mutanda** |  |  | In 1962, the desmids *Staurastrum*  and *Closterium*, and *Pediastrum* and *Coelastrum* dominated [1] |  | In 1990, *Ceratium* and a few *Microcystis* dominated. In 2001,  *Microcystis* was dominant [1] |
| **Mulehe** |  |  | In 1961, *Melosira*, *Synedra*, *Oscillatoria* and *Coelastrum* dominated [1] | In 1975, *Melosira* and *Microcystis* and *Pediastrum* [1] | In 1990,  *Microcystis* and *Ceratium* dominated [1] |
| **Zooplankton observations** | | | | | |
| **Bunyonyi** |  | In 1931, *Daphnia curvirostris*,  *D. laevis*, *Ceriodaphnia reticulata* and *Metadiaptomus aethiopicus* dominated [1] | In 1962, *Thermocyclops*  increased and replaced the earlier species.  The lake was rich in rotifers but dominated by *Keratella tropica* [2] | In 1975, no  *Metadiaptomus*, *Daphnia laevis* or *Ceriodaphnia reticulata* recorded from the lake [1] | In 1990, only one specimen of *D. curvirostris* was found among thousands of *Moina micrura*  and *Thermocyclops. Daphinia* sp. disappeared by 2001 [1] |
| **Mutanda** | *Daphnia spp*,  *Ceriodaphnia reticulata* and *Metadiaptomus aethiopicus* dominated [1] | *Daphnia spp*,  *Ceriodaphnia reticulata* and *Metadiaptomus aethiopicus* dominated [1] | Between 1962 and 1975, *Daphnia spp*,  *Ceriodaphnia reticulata* and *Metadiaptomus aethiopicus* disappeared [1]. In 1962, rotifers were sparse but dominated by *Tetramastix opoliensis* [2]. | | *Thermocyclops oblongatus*  and *Moina micrura*, dominated [1] |
| **Mulehe** | *Ceriodaphnia reticulata* dominated [1] | *Ceriodaphnia reticulata* dominated [1] | Between 1962  and 1975, *Ceriodaphnia reticulata* disappeared [1]. In 1962, the lake was dominated by *Synchaeta pectinata* [2] | |  |
| **Fish status from fishless to the invasive** | | | | | |
| **Bunyonyi** | Originally fishless, in 1919 the small catfish *Clarias*  *carsonii* was introduced [1] | In 1928, *Oreochromis niloticus* was introduced [1] | In 1958, *Oreochromis esculentus* and some  Lake Victoria *‘Haplochromis’* were introduced [1] |  |  |
| **Mutanda** | *Tilapia* sp. present [3] | | In 1960, *Macropterus salmoides* was introduced and coexisted with the *Tilapia* sp. that was present [3] | In 1968, *Macropterus salmoides* disappeared and *Tilapia* sp. declined [3]  In 1973, *Cyprinus carpio* was introduced although it declined by 1977 [3] | In 1991, the fishers resorted to harvesting *Clarias carsoni*, haplochomines and edible frogs (*Xenopus kigesiensis*) [3] |
| **Mulehe** | *Oreochromis* introduced [1] | | In 1962, Lake Mulehe had the most productive fishery due to the introduction of *Oreochromis* [1]  In 1962, common carp was introduced [3] | *Cyprinus carpio* were introduced in the lake, flourished for a few years and became scarce [1]. In 1973, massive fish kills occurred [3] | In 1983, another massive fish kill event happened that led to the decline of haplochromine [3] |
| **Burera** |  | Between 1926  and 1935, only a few *Clarias*  *liocephalus* were present [4] | In 1952, *Tilapia* were abundant, and *Haplochromis* and *Barbus* species seemed to have disappeared [4] |  | In 1988, *Tilapia, Haplochromis*, *C. liocephalus, and Amphilius uranoscopus* were common [4].  In 1991, *Rastrineobola argentea* was introduced [5] |
| **Ruhondo** | In 1907, *Barbus neumayeri* was the only fish present in the lake [4] | In the 1930s, *Tilapia nilotica* was introduced by the Belgian government In 1937, *Barbus microbarbis,* and  *Varicorhinus ruandae* were present [4] | In 1952, *Tilapia* abundant, *Haplochromis* and *Barbus* species seemed to have disappeared [4] | In 1980, *B. neumayeri* was rare in the lake, but common in a few small inflowing streams of the  Lake [4]. larger *Barbus* i.e. *Varicorhinus*  were no longer in existence [4] | In 1989, the Lake  Comprised of one *Haplochromis* species,  one *Tilapia* species*,* C. *liocephalus,* and a very small numbers of *B. neumayeri*  and *P. Multicolor* [4]*.*  In 2006, *Rastrineobola argentea* colonized Ruhondo [5] |
| ***Procambarus* first appearance** | | | | | |
| **Bunyonyi** |  |  |  | In 1966, crayfish was introduced [6] | In 2007, crayfish had well-established and abundant [6] |
| **Mutanda** |  |  |  |  | According to our current findings, crayfish is present in few shoreline localities |
| **Mulehe** |  |  |  |  | According to our current findings, crayfish was recorded from only one locality on R. Mucha draining the lake |
| **Burera** | In Rwanda, crayfish was introduction in Mukungwa valley as a biological control agent of water hyacinth but the year of introduction remains  un known [6] | | | | |
| **Ruhondo** |  |  |  |  |  |
| ***Xenopus* extinction and exchanged species** | | | | | |
| **Bunyonyi** |  | In 1925, *X. laevis bunyoniensis* (85%) dominated with *few X. wittei*  Between 1925 and 1935, only *X. laevis bunyoniensis* was present [1] |  | In 1972,  only *X. wittei* was present [1] |  |
| **Mutanda** |  | In 1934, *X. laevis bunyoniensis*  was present [1] | In 1969, *X. vestitus* dominated with very few *X. laevis bunyoniensis* [1] | In 1975, *X. laevis bunyoniensis* became too rare [1] |  |
| **Mulehe** |  | In 1934, *X. laevis bunyoniensis* dominated [1] |  | In 1975, *X. laevis bunyoniensis* disappeared, was replaced by *X. wittei* and  *X. vestitus* [1] |  |
| **Eutrophication events** | | | | | |
| **Bunyonyi** |  |  | In 1961, there was a high concentration of phosphates in the lake even at a depth of 35 m (93 µg l−1 PO4 P ) [1] | In 1972, the phosphate level was about 480 µg l−1 at 30 m and 610 µg l−1 at 37 m [1] | Between 2019 and 2020, based on trophic state index by Carlson (1977), the lake was classified as eutrophic [7] |
| **Mutanda** |  |  |  |  | 1998 and 1999, the lake was relatively eutrophic with cyanobacteria dominating with a low transparency-2.84m [8] |
| **Mulehe** |  |  |  |  | Between 1998 and 1999, Cyanobacteria dominated algal blooms, and the lake transparency was very low ~ (0.4 - 0.5)m [8]. In 2022, lake was eutrophic turbid [9] |
| **Burera** |  |  |  |  | In 2019, phosphorus and nitrogen levels (8.28mg/l Burera and 8.38mg/l Ruhondo ) exceeded Class III EPA standards hence eutrophication risk [10] |
| **Ruhondo** |  |  |  |  |  |

**References**

Green, J. (1965) ‘Zooplankton of Lakes Mutanda, Bunyonyi and Mulehe’, in *Proceedings of the Zoological Society of London*. Wiley Online Library, pp. 383–400. Available at: https://doi.org/https://doi.org/10.1111/j.1469-7998.1965.tb05189.x.

Green, J. (2009) ‘Nilotic lakes of the Western Rift’, in *The Nile: Origin, Environments, Limnology and Human Use*. Springer, pp. 263–286. Available at: https://doi.org/https://doi.org/10.1007/978-1-4020-9726-3_14.

Habimana, V. and Nsabimana, A. (2020) ‘Water Physico-Chemical Characteristics of the Lakes Burera and Ruhondo, Rwanda’, *Rwanda Journal of Engineering, Science, Technology and Environment*, 3(2). Available at: https://doi.org/10.4314/rjeste.v3i2.5.

Isumbisho, M. *et al.* (2011) ‘The feeding habit of the Cyprinidae Rastrineobola argentea in its new habitat, lakes Bulera and Ruhondo, two Rwandan lakes (Eastern Africa)’, *Knowledge and Management of Aquatic Ecosystems*, (403), p. 4. Available at: https://doi.org/https://doi.org/10.1051/kmae/2011044.

Kamanyi, J.R., Ndawula, L.M. and Magumba, M. (2000) ‘The history and fishery potential of Kisoro Minor Lakes￼ part two: Preliminary survey of Lakes Mutanda and Mulehe fisheries￼’.

Madzivanzira, T.C. *et al.* (2020) ‘A review of freshwater crayfish introductions in Africa’, *Reviews in Fisheries Science & Aquaculture*, 29(2), pp. 218–241.

Magumba, M.K. (2000) ‘Physical, chemical, algal composition and primary production in the four Kisoro minor lakes’.

Saturday, A. *et al.* (2023) ‘Spatial and temporal variations of trophic state conditions of Lake Bunyonyi, south-western Uganda’, *Applied Water Science*, 13(1), p. 7. Available at: https://doi.org/https://doi.org/10.1007/s13201-022-01816-y.

Saturday, A., Kangume, S. and Bamwerinde, W. (2023) ‘Content and dynamics of nutrients in the surface water of shallow Lake Mulehe in Kisoro District, South–western Uganda’, *Applied Water Science*, 13(7), p. 150. Available at: https://doi.org/https://doi.org/10.1007/s13201-023-01953-y.

De Vos, L., Snoeks, J. and van den Audenaerde, D.T. (1990) ‘The effects of tilapia introductions in Lake Luhondo, Rwanda’, *Environmental Biology of Fishes*, 27, pp. 303–308. Available at: https://doi.org/https://doi.org/10.1007/BF00002748.
