# Supplementary material for: Invaders taking over—Mollusc faunal change in volcanic barrier lakes of the Albertine Rift biodiversity hotspot
Source: PLoS One. 2026 Jun 30;21(6):e0352648. doi: 10.1371/journal.pone.0352648 (PMC13318018; doi:10.1371/journal.pone.0352648)
Supplement: S2 Table — (DOCX) [file pone.0352648.s010.docx]

S6 **Table.** Results of BLAST searches, indicating species, with voucher code, the associated BLAST hits with % similarity (in brackets), NCBI GenBank accession numbers and the countries of origin. UGSB – University of Giessen Systematics and Biodiversity collection. The three-letter code indicated for BLAST results indicates the country of origin: CHN – China, CMR – Cameroon, EGY – Egypt, IRN – Iran, JPN – Japan, KEN – Kenya, RWA – Rwanda, UGA – Uganda, USA - United States of America, ZWE - Zimbabwe

| **Family** | **Species** (morphological) | **Voucher no.** | **BLAST results** (COI) | **BLAST results** (16S) | **GenBank acc. numbers** | **Name added in GenBank** | **Location** |
| --- | --- | --- | --- | --- | --- | --- | --- |
| **GASTROPODA** |  |  |  |  |  |  |  |
| **Ampullariidae** | *Pila ovata* | UGSB 30043 | *Pila ovata*  (99.68%) EU274571.1 UGA |  | PZ305685 | *Pila ovata* | Lake Mulehe |
| **Bulinidae** | *Bulinus truncatus* | UGSB 29951 | *Bulinus truncatus* (99.12%) NC_060795.1 EGY |  | PZ305697 | *Bulinus truncatus* | Lake Bunyonyi |
|  | *Bulinus truncatus* | UGSB 29952 | *Bulinus truncatus* (100.00%) MN551578.1 RWA |  | PZ305697 | *Bulinus truncatus* | Lake Bunyonyi |
|  | *Bulinus* sp. | UGSB 29947 | *Bulinus truncatus* (99.12%) NC_060795.1 EGY |  | PZ305698 | *Bulinus truncatus* | Lake Mulehe |
|  | *Bulinus* sp. | UGSB 29948 | *Bulinus truncatus* (98.84%) NC_060795.1 EGY |  | PZ492956 | *Bulinus truncatus* | Lake Mulehe |
|  | *Bulinus* sp. | UGSB 29949 | *Bulinus truncatus* (99.12%) NC_060795.1 EGY |  | PZ305698 | *Bulinus truncatus* | Lake Mulehe |
|  | *Bulinus truncatus* | UGSB 29959 | *Bulinus truncatus* (98.84%) NC_060795.1 EGY |  | PZ305698 | *Bulinus truncatus* | Lake Mulehe |
|  | *Bulinus* sp. | UGSB 29966 | *Bulinus truncatus* (99.13%) NC_060795.1 EGY |  | PZ305698 | *Bulinus truncatus* | Lake Mulehe |
|  | *Bulinus* sp. | UGSB 29950 | *Bulinus truncatus* (99.26%) NC_060795.1 EGY |  | PZ512325 | *Bulinus truncatus* | Lake Mulehe |
|  | *Bulinus* sp. | UGSB 29960 | *Bulinus truncatus* (100.00%) PP510652.1 RWA |  | PZ305699 | *Bulinus truncatus* | Lake Mulehe |
|  | *Bulinus* sp. | UGSB 29961 | *Bulinus truncatus* (100.00%) PP510651.1 RWA |  | PZ502131 | *Bulinus truncatus* | Lake Mulehe |
|  | *Bulinus* sp. | UGSB 29962 | *Bulinus truncatus* (100.00%) PP510651.1 RWA |  | PZ502132 | *Bulinus truncatus* | Lake Mulehe |
|  | *Bulinus* sp. | UGSB 30676 | *Bulinus truncatus* (95.00%) MG759469.1 ZWE |  | PZ507407 | *Bulinus truncatus* | Lake Ruhondo |
|  | *Bulinus truncatus* | UGSB 30678 | *Bulinus truncatus* (99.00%) KJ157409.1 CMR |  | PZ507408 | *Bulinus truncatus* | Lake Ruhondo |
|  | *Bulinus truncatus* | UGSB 30679 | *Bulinus truncatus* (98.00%) KJ157409.1 CMR |  | PZ507409 | *Bulinus truncatus* | Lake Ruhondo |
|  | *Bulinus* sp. | UGSB 30680 | *Bulinus truncatus* (98.00%) MG759469.1 ZWE |  | PZ507410 | *Bulinus truncatus* | Lake Ruhondo |
| **Lymnaeidae** | *Radix* *natalensis* | UGSB 29953 | *Radix* sp. (99.24%) OP084764.1 UGA |  | PZ305686 | *Radix* *natalensis* | Lake Bunyonyi |
|  | *Radix* sp. | UGSB 29955 | *Radix natalensis* (98.78%) MN601427.1 KEN |  | PZ305687 | *Radix* *natalensis* | Lake Bunyonyi |
|  | *Radix* sp. | UGSB 29956 | *Radix* sp. (99.24%) OP084764.1 UGA |  | PZ305688 | *Radix* *natalensis* | Lake Bunyonyi |
|  | *Radix* sp. | UGSB 29971 | *Radix* sp. (99.24%) OP084764.1 UGA |  | PZ305694 | *Radix* *natalensis* | Lake Bunyonyi |
|  | *Radix* sp. | UGSB 29972 | *Radix* sp. (99.24%) OP084764.1 UGA |  | PZ305695 | *Radix* *natalensis* | Lake Bunyonyi |
|  | *Radix* sp. | UGSB 29957 | *Radix* sp. (99.24%) OP084764.1 UGA |  | PZ305689 | *Radix* *natalensis* | Lake Mulehe |
|  | *Radix* sp. | UGSB 29958 | *Radix* sp. (99.39%) OP084764.1 UGA |  | PZ305690 | *Radix* *natalensis* | Lake Mulehe |
|  | *Radix* sp. | UGSB 29964 | *Radix natalensis* (98.78%) MN601427.1 KEN |  | PZ305691 | *Radix* *natalensis* | Lake Mulehe |
|  | *Radix* sp. | UGSB 29968 | *Radix natalensis* (99.36%) PP510649.1 RWA |  | PZ305692 | *Radix* *natalensis* | Lake Mulehe |
|  | *Radix* sp. | UGSB 29970 | *Radix sp.* (99.39%) OP084764.1 UGA |  | PZ305693 | *Radix* *natalensis* | Lake Mutanda |
|  | *Radix sp.* | UGSB 29973 | *Radix* sp. (99.39%) OP084764.1 UGA |  | PZ305696 | *Radix* *natalensis* | Lake Mutanda |
|  | *Radix natalensis* | UGSB 28399 | *Radix* sp. (98.00%) OP084764.1 UGA |  | PP528798 | *Radix* *natalensis* | Lake Ruhondo |
|  | *Radix natalensis* | UGSB 28418 | *Radix* sp. (98.00%) OP084764.1 UGA |  | PP528791 | *Radix* *natalensis* | Lake Burera |
|  | *Radix natalensis* | UGSB 28432 | *Radix natalensis* (97.54%) PP510649.1 RWA |  | PP528808 | *Radix* *natalensis* | Lake Burera |
|  | *Radix natalensis* | UGSB 28446 | *Radix natalensis* (98.43%) PP510649.1 RWA |  | PP528790 | *Radix* *natalensis* | Lake Ruhondo |
| **Physidae** | *Physella acuta* | UGSB 29723 | *Physella acuta* (98.00%) OQ561521.1 USA |  | PZ514000 | *Physella acuta* | Lake Burera |
| **Planorbidae** | *Biomphalaria* sp. | UGSB 29934 | *Biomphalaria* cf. *pfeifferi* (98.92%) DQ084833.1 UGA |  | PZ485582 | *Biomphalaria* cf. *choanomphala* | Lake Bunyonyi |
|  | *Biomphalaria* sp. | UGSB 29935 | *Biomphalaria* cf. p*feifferi (*98.83%) DQ084833.1 UGA |  | PZ485583 | *Biomphalaria* cf. *choanomphala* | Lake Bunyonyi |
|  | *Biomphalaria* sp. | UGSB 29936 | *Biomphalaria* cf. p*feifferi* (98.89%) DQ084833.1 UGA |  | PZ485584 | *Biomphalaria* cf. *choanomphala* | Lake Bunyonyi |
|  | *Biomphalaria* sp. | UGSB 29937 | *Biomphalaria choanomphala* (98.86%) HM768906.1 UGA |  | PZ485585 | *Biomphalaria* cf. *choanomphala* | Lake Bunyonyi |
|  | *Biomphalaria* sp. | UGSB 29940 | *Biomphalaria choanomphala* (99.01%) HM768912.1 UGA |  | PZ485587 | *Biomphalaria* cf. *choanomphala* | Lake Bunyonyi |
|  | *Biomphalaria* sp. | UGSB 29944 | *Biomphalaria choanomphala* (99.18%) HM768937.1 UGA |  | PZ485590 | *Biomphalaria* cf. *choanomphala* | Lake Mulehe R. Mucha |
|  | *Biomphalaria* sp. | UGSB 29945 | *Biomphalaria choanomphala* (98.82%) HM768934.1 UGA |  | PZ485591 | *Biomphalaria* cf. *choanomphala* | Lake Mulehe |
|  | *Biomphalaria* sp. | UGSB 29938 | *Biomphalaria choanomphala* (99.18%) HM768937.1 UGA |  | PZ485586 | *Biomphalaria* cf. *choanomphala* | Lake Mutanda |
|  | *Biomphalaria* sp. | UGSB 29941 | *Biomphalaria sudanica* (99.02%) HM768904.1 UGA |  | PZ485588 | *Biomphalaria* cf. *choanomphala* | Lake Mutanda |
|  | *Biomphalaria* sp. | UGSB 29942 | *Biomphalaria sudanica* (99.08%) DQ084843.1 UGA |  | PZ485589 | *Biomphalaria* cf. *choanomphala* | Lake Mutanda |
|  | *Biomphalaria* cf. *pfeifferi* | UGSB 28400 | *Biomphalaria* cf. *pfeifferi* (97.00%) DQ084833.1 UGA |  | PZ507411 | *Biomphalaria* cf. *choanomphala* | Lake Ruhondo |
|  | *Biomphalaria* sp. | UGSB 28411 | *Biomphalaria pfeifferi* (97.00%) NC_038059.1 KEN |  | PZ507412 | *Biomphalaria* cf. *choanomphala* | Lake Ruhondo |
|  | *Segmentorbis* sp. | UGSB 30041 |  | *Polypylis* sp. (95.69%) LC428911.1 JPN | PZ500523 | *Segmentorbis* sp. | Lake Bunyonyi |
|  | *Segmentorbis* cf. *angustus* | UGSB 30131 | Planorbidae sp*.* (96.95%) LC491289.1 KEN |  | PZ485592 | *Segmentorbis* sp. | Lake Bunyonyi |
|  | *Segmentorbis* cf. *angustus* | UGSB 30132 | *Planorbidae* sp*.* (97.97%) LC491289.1 KEN |  | PZ485593 | *Segmentorbis* sp. | Lake Bunyonyi |
| **Thiaridae** | *Melanoides tuberculata* | UGSB 30058 | *Melanoides tuberculata* (99.22%) KT280409.1 IRN |  | PZ305682 | *Melanoides tuberculata* | Lake Bunyonyi |
|  | *Melanoides tuberculata* | UGSB 30123 | *Melanoides tuberculata* (99.15%) KT280409.1 IRN |  | PZ305683 | *Melanoides tuberculata* | Lake Bunyonyi |
|  | *Melanoides tuberculata* | UGSB 30124 | *Melanoides tuberculata* (99.41%) MZ321058.1 CHN |  | PZ305684 | *Melanoides tuberculata* | Lake Mulehe |
|  | *Melanoides tuberculata* | UGSB 30125 | *Melanoides tuberculata* (100.00%) MZ321058.1 CHN |  | PZ305684 | *Melanoides tuberculata* | Lake Mulehe |
| **Viviparidae** | *Bellamya* sp. | UGSB 29792 | *Bellamya* cf. *unicolor* (99.83%) JX489240.1 RWA |  | PZ507413 | *Bellamya* cf. *unicolor* | Lake Ruhondo |
| **BIVALVIA** |  |  |  |  |  |  |  |
| **Sphaeriidae** | *Euglesa* sp. I | UGSB 30050 |  | *Euglesa milium* (99.20%) | PZ500524 | *Euglesa* sp. I | Lake Bunyonyi  tributary |
|  | *Euglesa* sp. I | UGSB 30051 |  | *Euglesa milium* (99.20%) | PZ500525 | *Euglesa* sp. I | Lake Bunyonyi  tributary |
|  | *E.* cf*. keniana* | UGSB 30055 | *Euglesa keniana* (99.48%) |  | PZ489842 | *E.* cf*. keniana* | Lake Bunyonyi  tributary |
|  | *E.* cf*. keniana* | UGSB 30134 | *Euglesa keniana* (99.83%) |  | PZ489843 | *E.* cf*. keniana* | Lake Bunyonyi  tributary |
|  | *E.* cf*. keniana* | UGSB 30049 |  | *Euglesa keniana* (100.00%) | PZ500526 | *E.* cf*. keniana* | Lake Bunyonyi  tributary |
|  | *E.* cf*. keniana* | UGSB 30048 |  | *Euglesa keniana* (100.00%) | PZ500527 | *E.* cf*. keniana* | Lake Bunyonyi  tributary |
|  | *Euglesa* sp. II | UGSB 30056 |  | *Euglesa* cf. *pirothi* (98.94%)  *Euglesa* cf. *viridaria* (98.94%) | PZ500528 | *Euglesa* sp. II | Lake Bunyonyi  tributary |
|  | *Euglesa* sp. II | UGSB 30057 | *Euglesa pirothi* (97.38%)  *Euglesa viridaria* (97.20%) | *Euglesa* cf. *pirothi* (98.94%)  *Euglesa* cf. *viridria* (98.94%) | PZ500529 | *Euglesa* sp. II | Lake Bunyonyi  tributary |
|  | *Euglesa* sp. III | UGSB 30054 |  | *Euglesa* cf. *pirothi* (99.79%)  *Euglesa* cf. *viridria* (99.79%) | PZ500530 | *Euglesa* sp. III | Lake Bunyonyi  tributary |
|  | *Sphaerium* sp. | UGSB 30053 |  | *Sphaerium nucleus* (98.41%) | PZ500531 | *Sphaerium* sp. | Lake Bunyonyi  proper |
|  | *Sphaerium* sp. | UGSB 30133 | *Sphaerium corneum* (95.80%) |  | PZ489844 | *Sphaerium* sp. | Lake Bunyonyi  proper |
|  | *Sphaerium* sp. | UGSB 30671 | *Sphaerium* cf. *hartmanni* (92.00%) PP510684.1 RWA |  | PZ507403 | *Sphaerium* sp. | Lake Ruhondo |
|  | *Sphaerium* sp. | UGSB 30672 | *Sphaerium* cf. *hartmanni* (95.00%) PP510684.1 RWA |  | PZ507404 | *Sphaerium* sp. | Lake Ruhondo |
|  | *Sphaerium* sp. | UGSB 30681 9821 | *Sphaerium* cf. *hartmanni* (94.00%) PP510684.1 RWA |  | PZ507405 | *Sphaerium* sp. | Lake Ruhondo |
|  | *Sphaerium* sp. | UGSB 30682 | *Sphaerium* cf. *hartmanni* (94.00%) PP510684.1 RWA |  | PZ507406 | *Sphaerium* sp | Lake Ruhondo |
