# Supplementary material for: Invaders taking over—Mollusc faunal change in volcanic barrier lakes of the Albertine Rift biodiversity hotspot
Source: PLoS One. 2026 Jun 30;21(6):e0352648. doi: 10.1371/journal.pone.0352648 (PMC13318018; doi:10.1371/journal.pone.0352648)
Supplement: S3 Table — (DOCX) [file pone.0352648.s011.docx]

S7 Table. Characteristics of the sampled lakes and streams, including altitude, physical and chemical characteristics of the water, substrate type, utilisation and the degree of anthropogenic disturbance. The degree of anthropogenic disturbance was qualitatively rated into three levels using a comparative approach based on the frequency of human activities. Abbreviations: DO-Dissolved oxygen, TDS-Total Dissolved Solids, Temp-Temperature, EC-Electrical conductivity

| **Parameters** | **Rwanda** | |  | **Uganda** | | | |  |
| --- | --- | --- | --- | --- | --- | --- | --- | --- |
|  | **Burera** | **Ruhondo** |  | **Bunyonyi** | **Tributaries to Bunyonyi** | **Mutanda** | **Mulehe** | **P-value** |
| **Altitude**  (m) | 1862 | 1759 |  | 1948 | 1944.50±6.50 | 1786 | 1795 | _ |
| **DO**  (mg/l) | 6.81±0.87 | 6.16±1.25 |  | 1.74±0.65 | 1.49±0.03 | 2.28±0.92 | 1.93±0.25 | <0.01 |
| **pH** | 7.93±0.41 | 8.26±0.31 |  | 8.72±0.15 | 8.84±0.30 | 8.93±0.09 | 8.50±0.23 | 0.01 |
| **TDS**  (ppm) | 116.53±50.08 | 205.74±5.20 |  | 118.75±1.48 | 66.00±8.00 | 137.00±40.31 | 114.00±1.63 | <0.01 |
| **Temp**  (℃) | 23.26±1.52 | 22.99±0.49 |  | 24.54±2.11 | 18.37±1.23 | 22.82±1.16 | 21.57±1.40 | >0.01 |
| **EC**  (µs) | 232.86±100.92 | 411.59±10.58 |  | 231.75±9.28 | 115.00±11.00 | 263.00±67.18 | 217.67±0.47 | <0.01 |
| **Substrates type** | Detritus, stones, sapropel | Sand, sapropel, detritus |  | Vegetation, detritus, sapropel | Stones, Soil, Silt | Vegetation, Stones, Detritus, sapropel | Stones, Stand, Detritus, Vegetation |  |
| Utilisation | Fishing, boating, tourism, Agriculture | Fishing, boating, tourism, Agriculture |  | Settlement, Tourism, Agriculture | Agriculture | Settlement, Tourism, Agriculture | Agriculture, Tourism |  |
| **Level of disturbance** | Medium | High |  | Medium | High | Low | Medium |  |
