# Supplementary material for: Invaders taking over—Mollusc faunal change in volcanic barrier lakes of the Albertine Rift biodiversity hotspot
Source: PLoS One. 2026 Jun 30;21(6):e0352648. doi: 10.1371/journal.pone.0352648 (PMC13318018; doi:10.1371/journal.pone.0352648)
Supplement: S5 Table — (DOCX) [file pone.0352648.s013.docx]

**Table 1** Mollusc community data NMDS score values

| **Site** | **NMDS1** | **NMDS2** |
| --- | --- | --- |
| **RWA24.008** | -0.00793229 | -0.51346055 |
| **RWA24.013** | -0.14234595 | -0.43196218 |
| **RWA24.014** | -0.07645923 | -0.4255286 |
| **RWA24.015** | 0.31004912 | -0.1700452 |
| **RWA24.017** | -0.27478739 | -0.0746754 |
| **RWA24.018** | -0.15877785 | -0.5561729 |
| **RWA24.019** | -0.06991458 | -0.13663235 |
| **RWA24.020** | 0.81658174 | 0.62136494 |
| **RWA24.021** | 0.05806247 | 0.21767622 |
| **RWA24.022** | 0.64473474 | 0.76251312 |
| **RWA24.023** | -1.28091726 | -1.68936123 |
| **RWA24.024** | -0.1098711 | 0.09671865 |
| **RWA24.025** | 0.05366529 | 0.06943817 |
| **UGA23.004** | 1.11262477 | -0.57698908 |
| **UGA23.007** | -0.35418506 | 1.32799706 |
| **UGA23.008** | -1.86549984 | -1.03071426 |
| **UGA24.009** | -1.19878473 | 0.85500516 |
| **UGA23.014** | -0.35418506 | 1.32799706 |
| **UGA23.015** | 1.73344028 | -1.13075658 |
| **UGA23.010** | 0.19942076 | 0.34655915 |
| **UGA23.013** | -0.35418506 | 1.32799706 |
| **UGA24.008** | 1.31926623 | -0.21696826 |

**Table 2** Environmental variables data NMDS score values

| **Variable** | **NMDS1** | **NMDS2** |
| --- | --- | --- |
| **Altitude** | -0.8485963 | 0.2664751 |
| **DO** | -0.4703237 | 0.1410824 |
| **pH** | 0.4897703 | 0.7697493 |
| **Temp** | -0.7387882 | -0.4572101 |
| **EC** | 0.2688901 | -1.1792777 |
| **Detritus0** | 0.03774712 | -0.06897563 |
| **Detritus1** | -0.06605746 | 0.12070735 |
| **Settlement0** | 0.17816542 | 0.25697884 |
| **Settlement1** | -0.14847118 | -0.21414903 |
| **Agriculture0** | 0.13579941 | -0.22847487 |
| **Agriculture1** | -0.11316618 | 0.19039573 |
| **Procambarus0** | -0.01984602 | 0.09905996 |
| **Procambarus1** | 0.02866647 | -0.1430866 |
